# *Retrozymes* are a unique family of non-autonomous *retro*transposons with hammerhead ribo*zymes* that propagate in plants through circular RNAs

**DOI:** 10.1186/s13059-016-1002-4

**Published:** 2016-06-23

**Authors:** Amelia Cervera, Denisse Urbina, Marcos de la Peña

**Affiliations:** IBMCP (CSIC-UPV). C/Ingeniero Fausto Elio s/n, 46022 Valencia, Spain

**Keywords:** Circular RNA, LTR retrotransposons, Viroid, Satellite RNA

## Abstract

**Background:**

Catalytic RNAs, or ribozymes, are regarded as fossils of a prebiotic RNA world that have remained in the genomes of modern organisms. The simplest ribozymes are the small self-cleaving RNAs, like the hammerhead ribozyme, which have been historically considered biological oddities restricted to some RNA pathogens. Recent data, however, indicate that small self-cleaving ribozymes are widespread in genomes, although their functions are still unknown.

**Results:**

We reveal that hammerhead ribozyme sequences in plant genomes form part of a new family of small non-autonomous *retro*transposons with hammerhead ribo*zymes*, referred to as *retrozymes*. These elements contain two long terminal repeats of approximately 350 bp, each harbouring a hammerhead ribozyme that delimitates a variable region of 600–1000 bp with no coding capacity. Retrozymes are actively transcribed, which gives rise to heterogeneous linear and circular RNAs that accumulate differentially depending on the tissue or developmental stage of the plant. Genomic and transcriptomic retrozyme sequences are highly heterogeneous and share almost no sequence homology among species except the hammerhead ribozyme motif and two small conserved domains typical of Ty3-gypsy long terminal repeat retrotransposons. Moreover, we detected the presence of RNAs of both retrozyme polarities, which suggests events of independent RNA-RNA rolling-circle replication and evolution, similarly to that of infectious circular RNAs like viroids and viral satellite RNAs.

**Conclusions:**

Our work reveals that circular RNAs with hammerhead ribozymes are frequently occurring molecules in plant and, most likely, metazoan transcriptomes, which explains the ubiquity of these genomic ribozymes and suggests a feasible source for the emergence of circular RNA plant pathogens.

**Electronic supplementary material:**

The online version of this article (doi:10.1186/s13059-016-1002-4) contains supplementary material, which is available to authorized users.

## Background

**Fig. 1:**
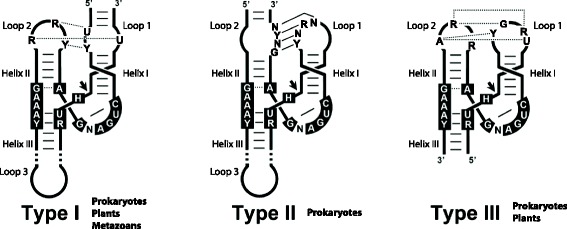
Representation of the three possible hammerhead ribozyme (HHR) topologies. The conserved nucleotides involved in the catalytic center are *boxed*. Conserved loop-loop interactions are also indicated. *Dotted* and *continuous* lines refer to non-canonical and Watson-Crick base pairs, respectively. The three HHR types have been reported in prokaryotic/phage genomes, whereas only types I and III have been described in plants. Metazoan genomes mostly show type I HHRs

The hypothesis of a prebiotic RNA world, where the first living organisms were based on RNA as both the genetic material and as catalyst [1–3], was strongly supported by the landmark discovery of ribozymes [4, 5]. It is thought that a few of those ancient ribozymes have remained in contemporary organisms performing key biological functions like the peptide bond formation by the ribosome [6], tRNA maturation by RNAse P [5] or mRNA splicing by the spliceosome [7], among others. In addition, there is an enigmatic group of small self-cleaving ribozymes that have been historically regarded as molecular oddities of some infectious RNA genomes, but recently have been found widespread in DNA genomes from all life kingdoms (for reviews see [8–10]). Among the few naturally occurring self-cleaving RNAs discovered, the hammerhead ribozyme (HHR) was the first and one of the best known members of the family. It is composed of a catalytic core of 15 conserved nucleotides surrounded by three double helixes (I to III), which adopt a γ-shaped fold where helix I interacts with helix II through tertiary interactions required for efficient in vivo activity [11–13]. Depending on the open-ended helix that connects the HHR motif to the flanking sequences, there are three possible circularly permuted forms named type I, II or III (Fig. 1). Originally described in small circRNA plant pathogens, like viral satellite RNAs [14] and viroids [15], the HHR catalyzes a self-cleavage transesterification reaction required during rolling-circle replication. A few HHRs were also described in DNA genomes of some unrelated eukaryotes like plants [16, 17], invertebrates [18, 19] and even vertebrates [20, 21], and were found mostly associated to repetitive DNAs. More recently, the widespread presence of HHRs in genomes from bacteria to eukaryotes has been reported [22–25], including human genomes [26], unveiling the HHR as an ubiquitous catalytic RNA motif [9, 10]. Similar results have been reported for other small small self-cleaving RNAs like the human hepatitis-δ [27] or twister ribozymes [28], which indicates that small ribozymes are frequent motifs encoded by DNA genomes. Although the precise biological functions of these genomic self-cleaving RNAs are yet unknown, their involvement in DNA retrotransposition would seem to be a frequent trend in eukaryotes [29, 30]. Retrotransposons are the major components of most eukaryotic genomes, especially in the plant kingdom, where long terminal repeat (LTR)retrotransposons can make up more than 70 % of the genome [31]. These retroelements encode the protein factors required for their own mobilization. Plant genomes, however, often contain many small non-autonomous LTR-retrotransposons, like the terminal-repeat retrotransposons in miniature (TRIMs) [32] or the small LTR-retrotransposons (SMARTs) [33], which do not encode any protein and whose mobility depends on autonomous LTR-retrotransposons. In general, all these retrotransposons and other mobile elements remain heavily silenced at the transcriptional level, and they are only expressed under certain conditions. In this work, following in silico, in vitro and in vivo approaches, we reveal that genomic HHRs in plants are part of an atypical family of non-autonomous LTR-retrotransposons that accumulate in the cell transcriptomes as abundant RNA circles.

## Results

### Genomic HHRs in plants are embedded in the LTRs of a new form of non-autonomous retroelement: retrozymes

The occurrence of HHRs has been previously reported in some plant genomes [9, 16, 17, 22]. In this work, we performed extensive bioinformatic searches for HHR motifs in plant genomes (see Methods section), which were mostly found in eudicots (42 species), notably among rosids, together with isolated examples in monocots, ferns and algae (Additional file 1). Type III HHRs were the most frequent motifs found, whereas only a few examples corresponded to type I HHRs. The number of ribozyme motifs detected per genome varied from the absence of any recognizable HHR in many species to more than 100 bona fide ribozymes in some others (Additional file 1).

As previously noticed [22], we confirmed that plant HHRs most frequently occur as isolated motifs, but also as close tandem repeats of two, three or, rarely, even four HHRs. Sequence repeats between HHR motifs were sized from 400 to 1000 bp, lacked any detectable protein-coding capacity and did not show clear sequence identity among different plant species. For each plant genome, isolated HHR motifs were usually found embedded within sequences of about 300–400 bp.

**Fig. 2:**
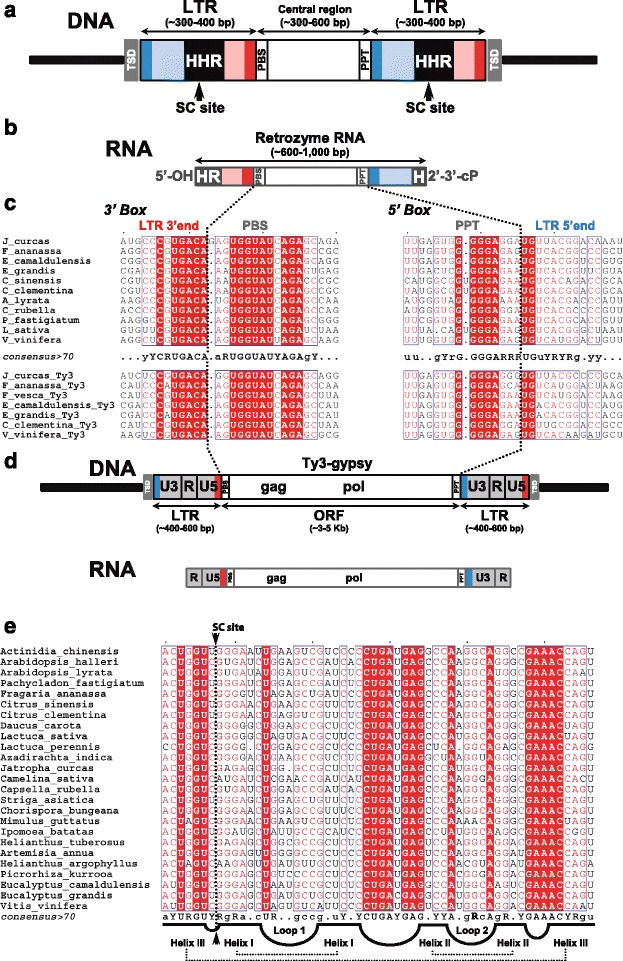
Sequence features of genomic retrozymes and Ty3-gypsy retrotransposons. **a** Schematic representation of a full genomic retrozyme element. Target site duplications (*TSDs*) delimiting the retrozyme are shown in *grey*. The 5′ and 3′ ends of the long terminal repeats (*LTRs*) are shown in *blue* and *red*, respectively, with the small conserved boxes highlighted in *darker colors*. The positions of the conserved primer binding site (*PBS*), the polypurine tract (*PPT*) and the hammerhead ribozymes (*HHR*) are indicated. The self-cleavage (*SC*) sites delimiting the retrozyme RNA are indicated with *arrows*. **b** Schematic representation of the transcribed and self-cleaved retrozyme RNA. **c** Alignment of the conserved 3′ and 5′ boxes (PBSs, PPTs and LTR ends) of representative retrozymes (*top*) and the equivalent regions of Ty3-gypsy retrotransposons (*bottom*). Consensus sequence of the motif is shown in the *middle*: totally conserved positions are shown as *uppercase letters* (either A, C, G, U, Y or R), whereas positions conserved in between 70–100 % of the sequences are shown as *lowercase letters*, and positions below 70 % in conservation are shown as *dots*. **d** Schematic representation of a genomic Ty3-gypsy retrotransposon (*top*) and its transcribed RNA (*bottom*). LTRs are shown in light *grey*. Small conserved 5′ and 3′ boxes are shown in *blue* and *red* respectively. Characteristic unique (U5, U3) and repeated (R) domains of the LTRs are indicated. The typical open reading frames of Ty3-gypsy retrotransposons are indicated as gag and pol. **e** Sequence alignment of selected HHRs from 25 plant species. The helixes and loops of the HHR are depicted at the bottom. Self-cleavage site is indicated with an *arrow*. Consensus sequences shown at the bottom were obtained as in panel **c**

Analysis of the elements containing tandem HHR copies showed that ribozymes were embedded in direct repeats of about 300–400 bp, which delimited a central region of about 300–600 bp without any coding potential (see Fig. 2a) [32]. These elements were flanked by target site duplications (TSDs) of 4 base pairs characteristic of LTR-retrotransposons. Most of these features are in common with those of TRIM and SMART retrotransposons (Additional file 2). However, given the peculiarities of these new retroelements, like the presence of catalytic RNA motifs in their LTRs, their slightly larger sizes and high sequence heterogeneity, as well as their singular transcriptional activity (see below), we named them retrozymes (after retroelements with hammerhead ribozymes).

We deduced that transcription of genomic retrozymes followed by self-processing through HHR motifs would result in RNA transposition intermediates (hereafter, retrozyme RNAs) of about 600–1000 nt, depending on the plant species (Fig. 2b). These retrozyme RNAs lack the characteristic repeated regions (R, Fig. 2b and d) of the transposition intermediates of LTR-retrotransposons required for retrotranscription of the full retroelement [34–37].

Homology of retrozyme sequences was evident between plants within the same genus, despite some clear heterogeneity. However, sequence identity was almost absent between retrozymes of less related plant species, with the exception of two small boxes of about 25 nt, referred to as 3′ and 5′ boxes (Fig. 2c), and the HHR motifs (Fig. 2e). To identify potential autonomous LTR-retrotransposons responsible for retrozyme mobilization, these small conserved boxes were used as queries to search against autonomous retroelements. We found that the conserved 3′ box in retrozymes is almost identical to the LTR 3′ end and the primer binding site (PBS, tRNA^Met^) of the Ty3-gypsy retrotransposons (Fig. 2c). The retrozyme 5′ box, in turn, is also very similar to the polypurine tract (PPT) and the LTR 5′ end of the same family of retrotransposons [38] (Fig. 2c). No other sequence similarities were detected between retrozymes and Ty3-gypsy or any other family of retrotransposons.

Overall, these data indicate that retrozymes constitute a new group of non-autonomous LTR retroelements that may use the machinery of plant Ty3-gypsy retrotransposons for their genomic mobilization in the same way as other non-autonomous retrotransposons do.

### Genomic retrozymes in the physic nut *Jatropha curcas*

*Jatropha curcas* or physic nut plant has a genome of about 410 Mb that has been recently sequenced [39–41]. In silico analysis of the available *J. curcas* sequences (70 % of the total genome) with RNAMotif revealed up to 30 bona fide type III HHRs, which showed some sequence heterogeneity for the same secondary structure (Additional file 3). Blast homology searches resulted in more than 70 HHR-like sequences (about 90 % identity for 90 nt), with 48 of these motifs occurring as tandem dimeric copies. These dimeric arrangements corresponded to 24 different retrozymes like the ones described above, whereas the rest of the HHRs motifs mostly corresponded to LTR sequences with no adjacent internal sequence (solo LTRs).

*J. curcas* retrozymes were flanked by TSDs of 4 bp with the consensus sequence WWRR (where W stands for A or T and R for a purine). LTRs were about 330 bp long, and the two HHR self-cleavage sites encompassed a non-coding region of 697–776 bp. At least four genomic retrozyme sequences were detected embedded within 18S rRNA gene sequences, whereas four others were found close (less than 2 kb) to LTR-retrotransposon sequences and the rest were detected within intergenic regions, in a similar way as described for other non-autonomous retroelements like TRIMs and SMARTs [32, 33].

To ascertain the activity of the HHRs contained in these retrozymes, in vitro transcription of a cloned *J. curcas* retrozyme fragment covering the 5′ LTR and the internal region was carried out. RNA self-cleaving activity was observed in the polarity containing the ribozyme motif (hereafter, the plus polarity of the retrozyme RNA) but not in the complementary (hereafter, the minus polarity) (Additional file 4). The amount of transcript processed by the HHR during transcription was 60 %, an efficiency similar to that reported for viroid and satellite HHRs [42].

### Different genomic retrozymes are transcribed, processed and accumulated as circRNAs in *J. curcas* tissues

**Fig. 3:**
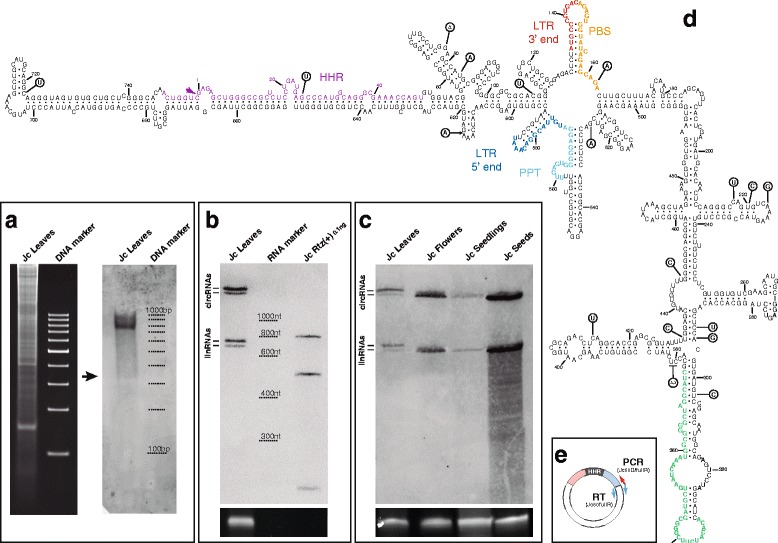
Retrozymes in the genome of the physic nut (*Jatropha curcas*). **a** Ethidium bromide staining of an RNA extract (~20 μg) from *J. curcas* leaves and a 100–1000 bp DNA ladder run in a 5 % native polyacrylamide gel (*left*) and its corresponding Northern blot hybridization (*right*) using as a probe a digoxigenin-labelled *J. curcas* retrozyme fragment of negative polarity. Approximate positions of the bands corresponding to the DNA marker are indicated on the Northern blot. **b** Northern blot analysis of an RNA extract (~30 μg) from *J. curcas* leaves carried out as in panel **a**. Doublet bands can be seen for linear as well as circular RNA molecules. 0.1 ng of a (+)RNA transcript of *J. curcas* retrozyme was included as a marker. Approximate positions of the bands corresponding to the RNA marker are indicated for clarity. **c** Northern blot analysis of RNA extracts (~30 μg each) from *J. curcas* leaves, flowers, seedlings and seeds. Samples were run in a 5 % denaturing polyacrylamide gel and detected as in panel **a**. The positions of the linear and circular RNAs are indicated. Ethidium bromide staining of the 5S rRNA band is shown at the *bottom* of panels **b** and **c** as a loading control. **d** Minimum free energy secondary structure prediction for a retrozyme RNA from *J. curcas* leaves (clone Jc_066, 753 nt). Numbering starts at the self-cleavage site of the HHR. The HHR motif is highlighted with *purple* letters, whereas the conserved 5′ and 3′ ends of the LTRs are shown in *blue* and *red*, respectively, and PPT and PBS regions in *cyan* and *orange*, respectively. A region of 45 nt usually absent in smaller variants is shown in *green* letters. Positions showing sequence heterogeneity among cloned variants from different plant tissues are indicated within *circles* (*triangle* corresponds to deleted nucleotides, whereas *arrows* correspond to inserted nucleotides). **e** Schematic representation of the circular retrozyme RNA and the position of the oligos used for RT and PCR experiments

Transcription is the first step in the replication cycle of retrotransposons and, under natural conditions, it is tightly repressed to avoid the mutational effects of transposon insertions in the host genome. To explore the transcriptional activity of retrozymes, RNA extracts from *J. curcas* leaves were initially analysed by native PAGE and Northern blot hybridization with probes complementary to the RNA intermediate of both polarities. A hybridization signal corresponding to the positive polarity of the retrozyme RNA (i.e. harbouring the HHR) was observed as a substantial band or bands around the 700 nt region (Fig. 3a). No band was detected for the negative polarity of the retrozyme (data not shown). These results indicate that at least some of the genomic retrozymes were transcribed and, likely, self-processed by the HHRs in leaves of *J. curcas*.

When the same RNA extracts from *J. curcas* leaves were analysed by denaturing PAGE followed by Northern blot hybridization, the bands in the 700 nt region appeared as a clear doublet (Fig. 3b), possibly corresponding to transcribed and HHR-processed RNAs from genomic retrozymes of different size. Interestingly, an additional doublet that did not appear in native gels was observed in denaturing gels with an apparent higher molecular weight (>3 kb) (Fig. 3b). This behaviour suggested the existence of a mixture of circular and linear RNAs that co-migrate in native gels, as has been observed during plant infection by pathogenic circRNAs such as viroids and virus satellites [43]. In order to confirm this finding, RNA samples were analysed by double PAGE: RNA extracts were first run in a native gel, and then the region of 600–800 nt was cut out and placed on a denaturing gel followed by Northern blot hybridization (Additional file 5A). This experiment confirmed that circular and linear RNAs co-migrated in the native gel and only became separated under denaturing conditions.

The transcriptional activity of genomic retrozymes in different tissues and developmental stages of *J. curcas* was analysed by Northern blot (Fig. 3c and Additional file 5B). RNA extracts from young seedlings and leaves showed the presence of two circular and their corresponding two linear bands. In flowers and seeds extracts, however, only the faster migrating circular and linear RNAs were observed. This result indicates that retrozymes are differentially transcribed in different tissues of *J. curcas*.

Purified circRNAs from *J. curcas* seeds, young seedlings and leaves were retrotranscribed, cloned and sequenced (Additional file 6). Two types of variants were detected in leaves, one of 753 nt approximately and a second one of about 708 nt, which are in agreement with the size estimated for the two linear bands detected by denaturing Northern blots. Prediction of minimum free energy secondary structures for the cloned retrozyme RNAs revealed a highly structured architecture with an elevated degree of self-complementarity (about 70 % of nucleotides are paired), similar to that reported for circRNA pathogens with HHRs [43] (Fig. 3d and Additional file 7). In the predicted structures, the LTR region adopts a long and stable hairpin structure with most of the HHR motif paired with a highly complementary sequence that prevents the hammerhead fold from forming and, consequently, its self-cleavage.

The obtained cDNA clones showed sequence variability between them (Fig. 3d), but also with respect to any of the genomic copies detected in the databases (Additional file 6). Such a high sequence heterogeneity together with the similarities between retrozyme and plant pathogenic RNAs (circular and highly structured RNA molecules, small size and presence of HHRs) suggested the possibility that retrozyme circRNAs may follow RNA-RNA replication through a rolling-circle mechanism similar to that described for viroids and viral satellite RNAs [44]. If that were the case, there would exist replication intermediates of negative polarity, either as circular or multimeric linear RNAs. To explore this possibility, we carried out RT-PCR experiments with adjacent primers outside of the LTRs to avoid the amplification of negative polarity RNAs resulting from transcription of genomic retrozyme copies (see Methods). Positive results were obtained with RNA extracts from *J. curcas* seeds (Additional file 8A). Northern blot analysis of RNA-enriched extracts from *J. curcas* tissues, however, did not reveal the presence of negative polarity RNAs of the retrozyme.

### Retrozyme-derived circRNAs accumulate to high levels in strawberry

HHR motifs have been previously reported in diverse genomic sequences of strawberry (*Fragaria x ananassa*) [22]. Thanks to the recently published genome of *F. ananassa* [45], our bioinformatic searches for HHRs revealed the presence of about 90 bona fide ribozyme motifs (Additional file 1) and up to 6 potential retrozyme RNAs of sizes ranging from 673 to 701 nt.

**Fig. 4:**
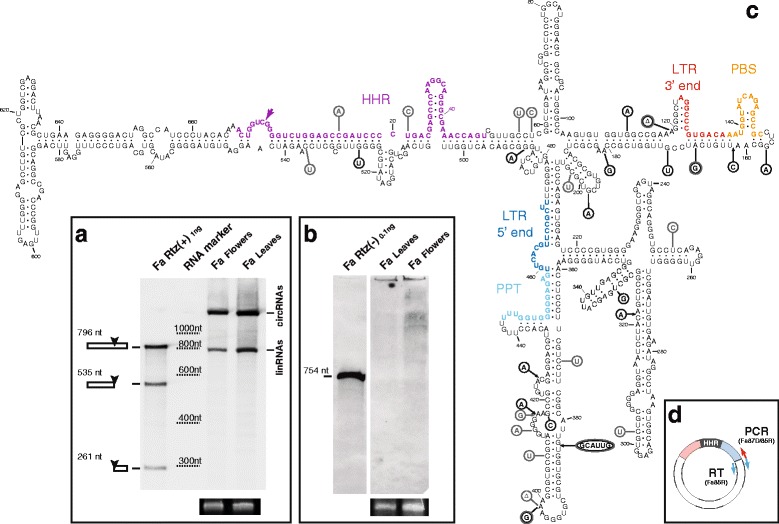
Retrozymes in the genome of the strawberry (*Fragaria ananassa*). **a** Northern blot analysis of RNA extracts (~20 μg) of *F. ananassa* flowers and leaves carried out as described in Fig. 3. One ng of a (+)RNA transcript of *F. ananassa* was included as a marker. The positions of the linear and circular RNAs are indicated. Approximate positions of the bands corresponding to the RNA marker are indicated for clarity. **b** Northern blot analysis of RNA extracts (~20 μg) of *F. ananassa* flowers and leaves run in a 5 % denaturing polyacrylamide gel and detected using a *F. ananassa* retrozyme fragment of the positive polarity as a probe. 0.1 ng of a (–)RNA transcript of *F. ananassa* was included. Ethidium bromide staining of the 5S rRNA band is shown at the bottom of panels **a** and **b** as a loading control. **c** Minimum free energy secondary structure prediction for a retrozyme RNA from *F. ananassa* leaves (clone Fa_049, 674 nt). Numbering, colors and natural sequence heterogeneity are shown as in Fig. 3. **d** Schematic representation of the circular retrozyme RNA and the position of the oligos used for RT and PCR experiments

In vitro transcription of a cloned genomic retrozyme fragment from *F. ananassa* carrying a single HHR motif showed a clear self-cleaving activity (48 % processed transcript) (Fig. 4a). Northern blot hybridization of RNA extracts revealed that retrozyme RNAs of positive polarity accumulated at high levels, with up to 0.1 ng of retrozyme RNAs per μg of total RNA from leaves (about 0.1 %), and slightly lower amounts from flowers (Fig. 4a). Northern blot analysis of the negative polarity did not reveal any clear band, but just a weak smear (Fig. 4b). However, in a similar way as found for *J. curcas* seeds, RT-PCR experiments with RNA extracts from *F. ananassa* leaves revealed the presence of retrozyme RNAs of negative polarity (Additional file 8B). Cloning and sequencing of the cDNAs from both positive and negative RNA polarities showed again a heterogeneous population of highly structured retrozyme RNAs (Fig. 4c).

To ascertain the presence of a mixture of circular and linear retrozyme RNAs in the plant, we carefully checked the migration properties of strawberry RNAs under native and denaturing conditions in the presence of appropriate markers obtained by in vitro transcription of a full genomic retrozyme of *F. ananassa* (Additional file 5C). The retrozyme RNA resulting from double self-cleavage (679 nt) was purified and circularized in vitro using a *Solanum melongena* tRNA ligase as previously described [46] (Additional file 5D). Purified linear and circularized retrozyme RNAs were run in a native PAGE together with an RNA extract of *F. ananassa*. Northern blot hybridization revealed a single band of about 700 nt in the three cases (Additional file 5E). When these three samples were run in a denaturing PAGE, the RNA extract of *F. ananassa* showed the typical duplet of bands, whereas the linear retrozyme RNA migrated as a 679-nt band and the circular RNA run with an apparent size of 3 kb. Circularized and linear RNA markers perfectly matched the two bands detected in the RNA extract, which confirms the presence of a mixed population of circular and linear retrozyme RNAs in the plant.

### Retrozymes in eucalyptus and citrus trees

In order to generalize the data obtained for the physic nut and strawberry, retrozymes of several woody plants were also investigated. Our bioinformatic analysis of the genomes of *Eucalyptus camaldulensis* [47] and *E. grandis* [48] detected more than 100 copies of bona fide HHRs in each of these genomes (Additional file 1). Dozens of ribozymes occurred in tandem copies of two, three and even four HHRs (Additional file 2D), suggesting large retrozyme RNAs of about 900–1050 bp.

**Fig. 5:**
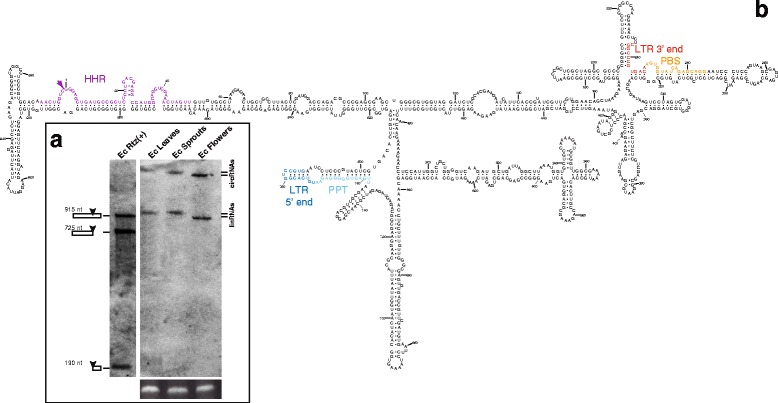
Retrozymes in the genome of the eucalyptus (*Eucalyptus camaldulensis*). **a** Northern blot analysis of RNAs (~20 μg) obtained from *E. camaldulensis* leaves, sprouts and flowers carried out as described in Fig. 3. 0.1 ng of a (+)RNA transcript of a genomic retrozyme from *E. camaldulensis* was included. The positions of the linear and circular RNAs are indicated. Ethidium bromide staining of the 7.5S rRNA band is shown at the bottom as a loading control. **b** Minimum free energy secondary structure prediction for a retrozyme RNA of 979 nt derived from a genomic retrozyme of *E. camaldulensis* (entry BADO01091291.1). Numbering and colors are shown as in Fig. 3

In vitro transcription of a cloned retrozyme fragment from *E. camaldulensis* showed clear levels (54 % processed transcript) of HHR self-cleavage (Fig. 5a). Northern blot analysis of RNA extracts from different *E. camaldulensis* tissues confirmed the presence of a retrozyme RNA of about 1 kb, although the band detected in flowers was of slightly lower molecular weight than the one found in leaves and young sprouts (Fig. 5a). The minimum free energy secondary structure of a genomic retrozyme RNA displayed a similar architecture to *J. curcas* and strawberry retrozymes: a long stable arm composed of the LTR sequence, and a ramified region of hairpins corresponding to the rest of the element (Fig. 5b).

We also analysed the genomic HHRs present in a number of citrus species. The genome of the sweet orange (*Citrus sinensis*, cv. Valencia) [49] only showed 10 bona fide HHRs, but up to 11 retrozyme-like elements able to encode putative retrozyme RNAs of 665–694 nt (Additional file 1). Many of the HHRs within putative retrozymes, however, showed punctual mutations that are expected to deeply affect their self-cleaving activity.

**Fig. 6:**
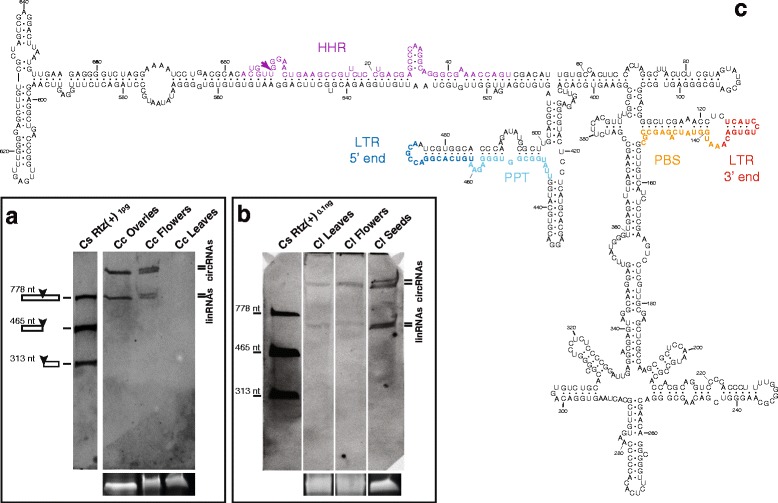
Retrozymes in the genome of different citrus trees. **a** Northern blot analysis of RNA extracts of *C. clementina* ovaries, flowers and leaves carried out as described in Fig. 3. 1 pg of a (+)RNA transcript of *C. sinensis* was included. **b** Northern blot analysis of RNA extracts of *C. limon* leaves, flowers and seeds carried out as described in Fig. 3. 0.1 ng of a (+)RNA transcript of *C. sinensis* was included. The positions of the linear and circular RNAs are indicated. Ethidium bromide staining of the 5S rRNA band is shown at the *bottom* of panels **a** and **b** as a loading control. **c** Minimum free energy secondary structure prediction for a retrozyme RNA of 689 nt derived from a genomic retrozyme of *Citrus clementina* (entry NW_006262201). Numbering and colors are shown as in Fig. 3

In vitro transcription of a retrozyme fragment carrying a bona fide HHR showed an intense RNA self-cleavage (about 75 % processed transcript) (Fig. 6a and b). However, RNA extracts from different *C. sinensis* tissues did not show the presence of circular or linear retrozyme RNAs, neither by Northern blot (detection limit of 0.1 pg) nor by RT-PCR analysis (data not shown). The genome of the related species *Citrus x clementina* revealed similar HHRs (up to 19 bona fide ribozymes) and 13 genomic retrozyme copies (Additional file 1). When RNA extracts of *C. clementina* were analysed by Northern blot, circular and linear RNAs were detected in ovaries and whole flowers, but not in leaves (Fig. 6a). In the case of lemon tissues (*Citrus x limon*), Northern blot analysis revealed the presence of circular and linear retrozyme RNAs in leaves, flowers and seeds (Fig. 6b). The minimum free energy secondary structure prediction for a genomic retrozyme RNA from *C. clementina* resulted again in a highly structured RNA (Fig. 6c).

Finally, comparative analysis of *C. clementina* (13 retrozymes) and *C. sinensis* (11 retrozymes) genomes gave us another indication of the mobile nature of retrozymes. Alignment of two orthologous genomic regions of around 5 kb (Additional file 9) showed that while *C. clementina* contains a typical retrozyme element flanked by a duplicated CTAT sequence (TSDs), the equivalent region in *C. sinensis* genome did not show any retrozyme sequence, but showed a single CTAT sequence at this particular position.

### Other putative retrozyme elements in plant and metazoan genomes

Our bioinformatic analyses revealed the presence of putative retrozymes in the genomes of more than 40 plant species (Additional file 1). Again, the retrozyme sequences in each plant genome showed a noticeable variability. Moreover, sequence identity of retrozymes from evolutionarily distant plant species was nearly absent, with the exception of the small conserved 5′ and 3′ boxes and the HHR motif (Fig. 2c and e). However, secondary structure prediction of minimal free energy for different plant retrozymes revealed a similar architecture, with a long arm corresponding to the LTR region harbouring the HHR in a blocked conformation, and a stable but more ramified structure corresponding to the rest of the RNA (Figs. 3d, 4c, 5b, 6c and Additional file 10).

**Fig. 7:**
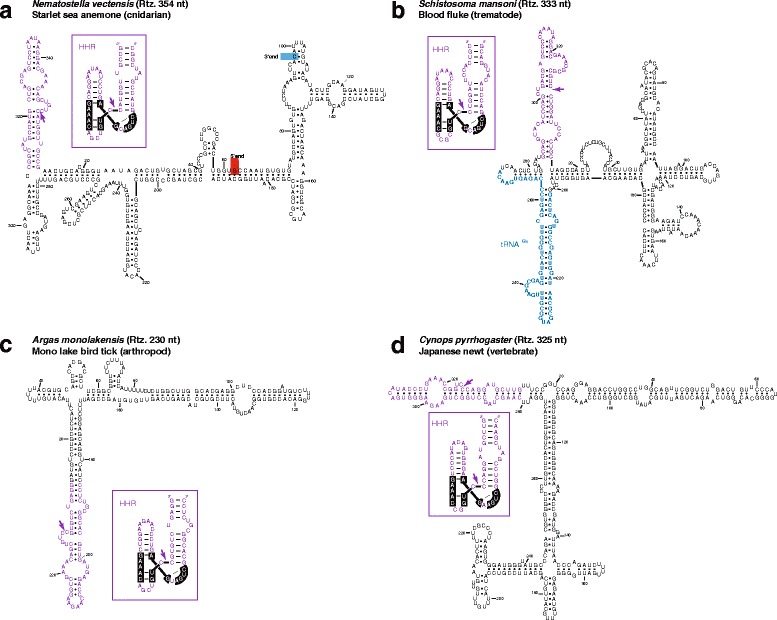
Minimum free energy secondary structure prediction for putative retrozyme RNAs deduced from different metazoan genomes: **a** the anemone *Nematostella vectensis*, **b** the trematode *Schistosoma mansoni* (tRNA^Glu^ sequence is shown in *blue*), **c** the tick *Argas monolakensis* and **d** the newt *Cynops pyrrhogaster. Insets* in each panel show the structure of the type I HHR. Similar representative examples of structures of metazoan retrozyme RNAs are shown in Additional file 11. HHR sequences are shown in *purple*, and the self-cleavage site is indicated with an *arrow*. Numbering starts at the self-cleavage site of the HHR

Genomic HHRs have also been reported previously in metazoans like newts [20], schistosomes [18] and cave crickets [19] and are widespread in a large set of animal genomes [9, 22, 24, 26]. Many of these type I HHR motifs occur as multimeric tandem repeats separated by a few hundred base pairs (about 150–400 bp) similar to those described above for plant retrozymes. However, our sequence analysis of metazoan repeats with HHRs did not detect any PBS or PPT motif, with the exception of a full tRNA^Gln^ in the case of *Schistosome* repeats [50]. Moreover, we found that *Nematostella vectensis* repeats were flanked by large TSDs (15 bp) similar to those described in the mobilization of long interspersed element (LINE) retrotransposons [51]. We also noticed that the monomeric RNA intermediates resulting from transcription and HHR processing of metazoan repeats show highly stable secondary structures (Fig. 7 and Additional file 11) similar to those found for plant retrozymes. We are aware that in silico RNA secondary structure prediction, especially for large RNA molecules, is not an accurate determination of the in vivo structure of the molecule. However, the stability and degree of self-complementarity deduced for these RNAs (above 70 % of the nucleotides are double-stranded) is much higher than the one observed for an RNA without any selection pressure on its secondary structure.

## Discussion

In this work, we have described the retrozymes, a new and atypical group of non-autonomous retroelements with self-cleaving ribozymes. At the genomic level, retrozymes highly resemble other small non-autonomous LTR-retrotransposons of plants like TRIMs [32] and SMARTs [33] (Fig. 2 and Additional file 2), but differ in some peculiarities that make them a unique class of retroelements. As non-autonomous retrotransposons, retrozymes do not show protein-coding regions but, in contrast, do encode active self-cleaving HHR motifs in their LTRs. These ribozymes catalyze the self-processing of the retrotransposon RNA intermediate, which accumulates in vivo as circular and linear non-coding RNAs of the precise size encompassed by the HHRs.

Genomic retrozymes show a patchy distribution among plants, occurring numerously in different species, but being absent in some others. For example, the eggplant (*Solanum melongena*) contains more than 150 HHRs and 18 different retrozymes, whereas the genomes of related *Solanum* species, like tomato or potato, do not show a single example. An illuminating case is found in the cassava genomes [52]. There are 34 full retrozymes in the wild variety (*Manihot esculenta* ssp. *flabellifolia*) but only 9 retrozyme copies in the genome of the domesticated one (*M. esculenta Crantz*), which suggests a negative selection pressure over these retroelements during plant domestication.

Another prominent feature of retrozymes is the high accumulation levels of heterogeneous circular and linear RNA intermediates in most of the plant tissues analysed. Retrotransposons are mostly quiescent in somatic cells, but activate under different stress conditions [38, 53, 54]. Our results indicate that, under natural conditions, some of the genomic retrozymes are either actively transcribed or weakly transcribed into highly stable covalently closed RNA circles. There is even the intriguing possibility that these circRNAs may undergo autonomous replication by plant polymerases as suggested by (1) the evident similarity of retrozymes with small circRNA pathogens with ribozymes (Additional file 6) [43], (2) the presence of multimeric retrozyme RNAs of the opposite polarity (Additional file 8) and (3) the observed sequence heterogeneity at RNA level (Additional file 7) indicative of replication events by error-prone RNA polymerases [44]. However, other explanations different from RNA replication are also possible, like a genomic origin of the minus RNAs, or sequence heterogeneity due to RNA hyperediting like that observed for some intronic circRNAs in animals [55]. Future research will be required to clarify these observations.

**Fig. 8:**
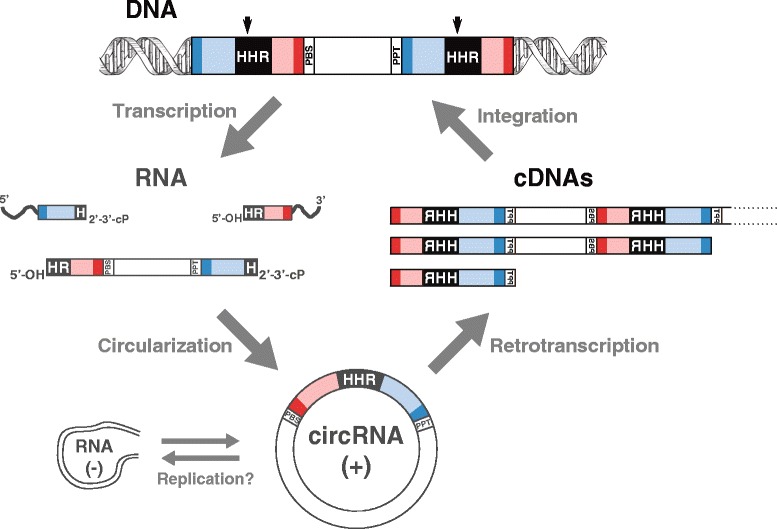
Model of the life cycle of retrozymes. A full genomic retrozyme containing at least two HHRs (*top*) is transcribed. The resulting RNA would self-process through the HHRs to give a linear RNA with 5′-OH and 2′-3′-cyclic phosphate ends, which would be circularized. The resulting circRNA(+) would be template for either endogenous RNA polymerases (replication cycles) and/or retrotranscriptases encoded by LTR-retrotransposons. In the latter case, the cDNAs resulting from retrotranscription of a circular RNA template could have different lengths (solo LTR, monomeric or multimeric) depending on the processivity of the retrotranscriptase. Finally, the machinery of the LTR-retrotransposon would integrate the retrozyme DNAs at a new genomic location

Regarding the retrotransposition mechanism of retrozymes, the most plausible model would involve the circRNAs as the final template for retrotranscription, whereas linear retrozyme RNAs would just be intermediaries and/or by-products of the circRNAs. This assumption is based on what we know about the retrotranscription of LTR retroelements, where the RNA template is a linear RNA carrying a characteristic repeated domain (R) at both ends that is necessary for 5′ to 3′ strand transfer and retrotranscription completion (Fig. 2d) [34, 37]. In retrozyme RNAs, processing by the self-cleaving ribozymes in the two LTRs produces a linear RNA with no repeated R domain (Fig. 2b). However, and as summarized in Fig. 8, covalent circularization of the self-cleaved RNAs through either the HHR itself or a host RNA ligase factor [43] would result in stable circRNAs able to bind a tRNA through their PBS. Retrotransposon-encoded retrotranscriptases could then be able to produce cDNAs of different lengths thanks to the circular nature of the RNA template (either solo LTR, full or multimeric retrozymes) (see Additional file 2). Finally, the resulting cDNAs would be integrated in new genomic locations through the machinery of the autonomous LTR-retrotransposons (Fig. 8).

## Conclusion

In summary, our work reveals that genome-encoded circRNAs carrying a self-cleaving ribozyme like the HHR are frequent molecules in plant transcriptomes, and constitute a feasible source for the origin of some virus satellites and viroids. In this regard, host RNAs derived from Ty3-gypsy retroelements are known to be efficiently encapsidated by the coat protein of a plant virus [56], and also Ty1-copia retrotransposons have already been proposed as the origin of non-HHR viroids of the family *Pospiviroidae* [57]. Finally, a plethora of splicing-derived circRNAs with diverse biological functions have been recently reported in eukaryotes [58–64] and, consequently, future research will be focused on deciphering the possible roles and biotechnological applications of genome-encoded circRNAs with HHRs.

## Methods

### Bioinformatics

RNAMotif [65] was used for the detection of canonical type I and type III HHR motifs in DNA sequences and whole genomes previously downloaded from public repositories (phytozome.jgi.doe.gov, ftp.ncbi.nlm.nih.gov). The hits obtained were inspected for the presence of tertiary interactions between helixes I and II to ensure they were bona fide HHRs. Sequence homology searches through BLAST, BLASTX [66] and BLAT [67] tools were carried out against sequences of the GenBank and Whole Genome Shotgun (WGS) sequence databases. Sequence alignments were performed with ClustalX and Jalview software [68]. Secondary RNA structures of minimum free energy were calculated with the RNAfold program from the ViennaRNA Package [69] and depicted with RnaViz [70].

### DNA and RNA extraction

DNA from leaves of the different plants analysed was extracted following the CTAB-chloroform protocol [71] with some modifications. Briefly, the leaves were homogenized in CTAB extraction buffer with a Polytron (Kinematica) homogenator and incubated at 60 °C for 60 min. The homogenate was mixed with an equal volume of chloroform and isoamyl alcohol (24:1 v/v). DNA in the aqueous phase was precipitated with 2.5 volumes of 100 % ethanol and 0.1 volume of 3 M sodium acetate, dissolved in MilliQ water and quantified in a NanoDrop 1000 Spectrophotometer (Thermo Fisher Scientific).

For RNA extractions, the CTAB-chloroform method [72] was used with some modifications, followed by purification with silica [73]. Briefly, the frozen material (seeds, seedlings, flowers, sprouts or leaves) was homogenized in CTAB extraction buffer with a Polytron homogenator (Kinematica) and incubated at 65 °C for 30 min. The homogenate was extracted twice with an equal volume of chloroform and isoamyl alcohol (24:1 v/v). RNA in the aqueous phase was purified by adding 0.5 volume of 100 % ethanol, one volume of 6 M NaI and 0.175 volume of 100 % SiO2 (pH 2). The slurry was incubated for 30 min at room temperature and then washed four times with a buffer containing 10 mM Tris-HCl, 0.5 mM EDTA, 50 mM NaCl and 50 % ethanol. The RNA was eluted in MilliQ water by incubating 4 min at 70 °C, and finally it was concentrated by precipitation with ethanol and quantified as described above.

*J. curcas* seeds of two different origins (called Jc India and Jc Malaysia) were provided by SLF seeds (Dehradun, UL 248002 India). Molecular analyses were performed with material obtained from Jc India seeds, with the exception of those analyses shown for Jc Malaysia in Additional files 5B and 6.

### PCR, RT-PCR and molecular cloning of retrozyme fragments

Genomic retrozyme fragments containing one of the HHRs and one LTR sequence plus the central variable region were amplified by PCR. The proofreading enzyme PrimeSTAR HS DNA Polymerase (Takara) was used following the manufacturer’s instructions, together with adjacent degenerate primers designed to target conserved retrozyme regions (Additional file 12). Amplification products of the adequate size were extracted from native 5 % PAGE gel slices with phenol:chloroform:isoamyl alcohol (25:24:1) and concentrated by ethanol precipitation as described above. The purified amplicons were inserted between the *Xba*I and *Bam*HI restriction sites of the plasmid pBlueScript KS+, and were sequenced automatically with an ABI Prism DNA sequencer (Perkin-Elmer). The resulting plasmids were used for analysis of ribozyme self-cleavage and probe synthesis for Northern blot.

Retrozyme RNAs, of both positive and negative polarity, were reverse-transcribed and PCR-amplified with divergent (adjacent and facing away from each other) primers (Additional file 12). RNA extracts were run in native 5 % polyacrylamide gels with 1× TAE, and a gel section of the appropriate retrozyme size was excised. RNA was purified from gel slices by phenol extraction and ethanol precipitation, and was digested with DNaseI (Roche Diagnostics GmbH). The enriched RNA extracts were used for reverse transcription (typically 100 ng RNA in a 20 μl reaction with SuperScript II, Invitrogen) and PCR (5 μl of retrotranscription products in a 50 μl reaction with PrimeSTAR HS DNA Polymerase), both performed following the instructions of the manufacturers. Amplicons of the adequate size were purified, cloned and sequenced as described above.

### Analysis of ribozyme self-cleavage and riboprobe synthesis

Retrozyme RNAfragments harbouring one HHR motif were synthesized by in vitro run-off transcription of pBlueScript KS+ plasmids containing the corresponding retrozyme insert previously linearized with *Eco*RI (for T7 RNA polymerase) or *Xba*I (for T3 RNA polymerase). For ribozyme self-cleavage analysis, transcription reactions contained: 40 mM Tris-HCl (pH 8), 6 mM MgCl_2_, 2 mM spermidine, 0.5 mg/ml RNase-free bovine serum albumin, 0.1 % Triton X-100, 10 mM dithiothreitol, 1 mM each of ATP, CTP and GTP, 0.1 mM UTP plus 0.5 μCi/μl [α-32P]UTP, 0.4 U/μl of porcine liver ribonuclease inhibitor (Takara), 20 ng/μl of plasmid DNA and 4 U/μl of T7 (Takara) or T3 (Roche Diagnostics GmbH) RNA polymerases. After incubation at 37 °C for 1–2 h, products were fractionated by polyacrylamide gel electrophoresis (PAGE) in 5 % gels with 8 M urea, and detected by phosphorimaging (FLA-5100 phosphorimager with BAS-MP 2040S imaging plates, Fujifilm). For the synthesis of DIG-labelled riboprobes of positive and negative polarity, transcription reactions were carried out in the same conditions as described above, except that radiolabelled UTP was replaced by 0.5 mM digoxigenin-11-UTP (Roche Diagnostics GmbH) and reactions were incubated at 37 °C for 4 h.

### Northern blot hybridization

For Northern blot analysis, from 5 up to 100 μg of purified RNA from different plant tissues were examined in 5 % polyacrylamide gels containing 8 M urea and 1× TBE (89 mM Tris/89 mM boric acid/2.5 mM EDTA, pH 8.3). For double PAGEs, nucleic acids enriched in RNAs of the appropriate retrozyme size were obtained by cutting a section from nondenaturing 5 % polyacrylamide gels. These RNAs were examined in denaturing 5 % polyacrylamide gels containing 8 M urea and 0.25× TBE (22.5 mM Tris/22.5 mM boric acid/2.5 mM EDTA, pH 8.3). After ethidium bromide staining, RNAs were electroblotted to nylon membranes (Amersham Hybond-N, GE Healthcare) and UV-fixed with a crosslinker (UVC 500, Hoefer). Prehybridization, hybridization (at 68 °C in 50 % formamide for 16 h) and washing (twice with 0.1× SSC at 68 °C for 15 min) was done following the instructions of the manufacturer (GE Healthcare). The DIG-labelled probes were detected with an anti-digoxigenin antibody conjugated with alkaline phosphatase (anti-digoxigenin-AP Fab fragments, 1:10^4^ dilution in blocking solution; Roche Diagnostics GmbH). The chemiluminiscence produced in the presence of the substrate CDP-*Star* (1:200 dilution in 0.1 M Tris-Cl, 0.1 M NaCl, pH 9.5; Roche Diagnostics GmbH) was finally visualized in a LAS-3000 Imaging System (Fujifilm).

### Cloning, transcription and circularization of a full genomic retrozyme from *F. ananassa*

The genomic retrozyme with the highest sequence homology to most of the cloned *F. ananassa* retrozyme RNAs [GenBank:BATT01039028.1:c14263-13183] was amplified by PCR using primers designed to bind outside the sequence of the retrozyme (Fa92D and Fa92R, Additional file 12). The PCR product was cloned in the *Xba*I and *Bam*HI sites of pBlueScript KS+, and the resulting plasmid was *Xba*I-linearized for run-off transcription with T3 RNA polymerase. The full uncleaved transcript and the retrozyme RNA resulting from self-cleavage at both HHR motifs were fractioned in 5 % polyacrylamide gels containing 8 M urea and 1× TBE, and extracted from gel slices as described above.

The retrozyme RNA, with 5′-hydroxyl and 2,3′-phosphodiester termini, was circularized using the chloroplastic isoform of a tRNA ligase from *Solanum melongena* [46], kindly provided by Drs. J. A. Daròs and R. Flores. The circularization reactions contained 1 μg of retrozyme RNA, about 1 μg of purified tRNA ligase, 50 mM Tris-HCl (pH 8), 50 mM KCl, 4 mM MgCl_2_, 5 mM DTT and 1 mM ATP, in a final volume of 50 μl. Reactions were incubated for 2 h at 30 °C and stopped by phenol extraction followed by ethanol precipitation. The circularized RNA was then separated in 5 % polyacrylamide gels containing 8 M urea and 1× TBE, and phenol-extracted from gel slices.

## Abbreviations

circRNA, circular RNA; HHR, hammerhead ribozyme; LINE, long interspersed element; LTR, long terminal repeat; MITE, miniature inverted-repeat transposable element; PBS, primer binding site; PPT, polypurine tract; RT, retrotranscriptase; SINE, small interspersed nucleotide element; SMART, small LTR-retrotransposon; TRIM, terminal-repeat retrotransposon in miniature; TSD, target site duplication

## Additional files

Additional file 1: Table S1. Bioinformatic compilation of putative HHRs and retrozymes found in Phytozome v10.0 and GenBank (15 Aug 2014) sequence databases of plants (see Methods section for more information). (PDF 76 kb) Additional file 2: Schematic representation of small plant non-autonomous LTR-retrotransposons. **A**: TRIM (*top*) and SMART (*bottom*) retroelements. **B**: Truncated solo-LTR. **C**: Full-copy retrozyme. **D**: Multimeric retrozyme. LTRs are shown in *blue* and the approximated sizes of the different elements and regions are indicated. (PDF 86 kb) Additional file 3: Hammerhead ribozymes in the genome of *Jatropha curcas.* **A**: Sequence alignment of the 30 bona fide HHRs detected in the genome of *J. curcas* (Bioprojects: PRJNA63485 and PRJNA279873). Only 5 out of the 30 HHRs show identical sequences, whereas the other 25 motifs showed one or more changes.** B**: Secondary structure of the *J. curcas* HHRs. Variable positions are indicated within *circles* (single changes) or *boxes* (covariations). Conserved tertiary interactions between loops 1 and 2 are shown with *dotted lines*. (PDF 128 kb) Additional file 4: Catalytic functionality of *J. curcas* hammerhead ribozymes. Schematic representation (*top*) and autoradiography (*bottom*) of run-off transcriptions in the presence of [α-32P]-UTP of the positive and negative polarities of a genomic retrozyme fragment from *Jatropha curcas*. The transcribed RNAs covered approximately from position 574 to position 573 of the circular RNA depicted in Fig. 3a, and were separated by denaturing 5 % PAGE. Quantification of the bands indicated that 60 % of the transcript was processed by the ribozyme. The position of T7 and T3 RNA promoters, primer binding site (*PBS*), polypurine tract (*PPT*) and HHR self-cleavage site (*arrowhead*) are indicated. (PDF 108 kb) Additional file 5: Circular and linear retrozyme RNAs in *J. curcas* and *F. ananassa*. **A**: Double PAGE analysis of an RNA extract (~20 μg) from *J. curcas* leaves. A gel stripe from a 5 % native polyacrylamide gel containing RNAs of 600–900 nt (*left*) was cut out and run on top of a second 5 % denaturing polyacrylamide gel (*center*). The corresponding Northern blot (*right*), using a digoxigenin-labelled *J. curcas* retrozyme fragment as a probe, revealed the presence of both circular and linear RNA forms. **B**: Northern blot analysis of RNA extracts (~30 μg each) from *J. curcas* leaves, young seedlings and seeds. Samples were run on a 5 % denaturing PAGE and were detected using the same probe as in panel A. **C**: An in vitro transcription of a previously cloned full genomic retrozyme of *F. ananassa* was run on a 5 % denaturing PAGE. The RNAs corresponding to the uncleaved full RNA (1134 nt) and double self-cleaved retrozyme RNA (679 nt) were cut out and purified (marked in *red*). **D**: Purified retrozyme RNA (679 nt) was circularized with a tRNA ligase for 2 h and run on a 5 % denaturing PAGE. The circularized RNA (*upper band*) was cut out and purified. **E**: Northern blot hybridization of a *F. ananassa* RNA extract (~30 μg) and previously purified RNA markers (linear, circular and uncleaved full retrozyme RNAs, ~1 ng each) run on a 5 % native PAGE. F: Northern blot hybridization of the same RNA samples as in panel E run on a 5 % denaturing PAGE. Ethidium bromide staining of the 5S rRNA is shown at the *bottom* as a loading control. (PDF 728 kb) Additional file 6: Sequence variability of genomic retrozymes and retrozyme RNAs. Sequence alignment of the 24 different genomic retrozymes found in *J. curcas* (names in *black*) against 16 selected clones of circRNAs from *J. curcas* tissues (names in *red*; 8 clones for each of the two major variants detected). Only the nucleotides encompassed by the HHR self-cleavage sites (equivalent to an HHR-processed retrozyme RNA) were used in the alignment. SM and SI correspond to circRNAs from *J. curcas* seeds, whereas HM and HI were obtained from leaves, and PM and PI from young seedlings. Based on their highest degree of similarity with the cloned RNAs, two genomic retrozyme sequences (at the *top* and at the *9th position* of the alignment) were chosen as the tentative parental elements. For clarity, the full sequence of the two tentative parental retrozymes is shown, whereas for all the other sequences, only the non-conserved positions are depicted. (PDF 110 kb) Additional file 7: Minimum free energy secondary structure predictions for the viral satellite RNA sTRSV (*top,* entry M14879.1) and the viroids ELVd (*middle,* entry AJ536612.1) and CChMVd (*bottom,* entry AJ878085.1). HHR sequences are shown in *purple* (positive polarity) and *grey* (negative polarity). Self-cleavage sites are indicated with *arrows*. Numbering starts at the self-cleavage site of the positive polarity HHR. (PDF 86 kb) Additional file 8: RT-PCR amplification of negative polarity retrozyme RNAs from *J. curcas* and *F. ananassa*. **A**: RT-PCR experiment carried out with RNA extracts from *J. curcas* seeds using the direct primer Jc60D for RT to make a cDNA of the minus polarity, and the adjacent primers Jc60D and Jc60fullR (*left*) or Jc77D and Jc77R (*right*) for the PCRs (see Additional file 12). **B**: RT-PCR experiments performed with two different RNA extracts from *F. ananassa* leaves using the direct primer Fa87D for RT to make a cDNA of the minus polarity, and the adjacent primers Fa87D and Fa85R for PCR (see Additional file 12). PCR products and 100 bp DNA marker were separated by native 5 % PAGEs and stained with ethidium bromide. Controls (RT-) were the same RT-PCR experiments done without adding retrotranscriptase, whereas Controls (PCR) were PCRs done without any template. Schematic representations of a full genomic retrozyme and the positions of the oligos used for RT and PCR experiments are shown below each gel picture. (PDF 649 kb) Additional file 9: A retrozyme insertion in the genome of *C. clementina* is absent from the orthologous region in the genome of *C. sinensis.* **A**: Comparative analysis of two orthologous genomic regions of around 5 kb from *C. clementina* and *C. sinensis* revealed the presence in the former but not in the latter genome of a typical retrozyme element (not to scale) flanked by the target site duplications (TSDs, in *grey* letters). The position of hammerhead ribozymes, the primer binding site and the polypurine tract are indicated. **B**: Sequence alignment of the two orthologous genomic regions of *C. clementina* and *C. sinensis*. Differences between both sequences are highlighted in *red*. The sequence corresponding to the genomic retrozyme is *underlined*. (PDF 118 kb) Additional file 10: Minimum free energy secondary structure prediction of five plant circRNA retrozymes deduced from the corresponding genomic retrozyme sequences (GenBank entries are indicated *below* the species names). HHR sequences are shown in *purple* letters and the self-cleavage site is indicated with an *arrow*. Numbering starts at the self-cleavage site of the HHR. (PDF 100 kb) Additional file 11: Minimum free energy secondary structure prediction of putative retrozyme RNAs of metazoan genomes from different phyla, like **A**: rotifers, **B**: corals, **C**: arthropods, **D**: mollusks and **E**: vertebrates, deduced from the corresponding sequence repeats with HHRs. HHR sequences are shown in *purple* letters and the self-cleavage sites are indicated with an *arrow*. Numbering starts after the HHR self-cleavage site. (PDF 104 kb) Additional file 12: Table S2. Oligonucleotide compilation. (PDF 58 kb)
