# Erratum to: *Retrozymes* are a unique family of non-autonomous *retro*transposons with hammerhead ribo*zymes* that propagate in plants through circular RNAs

**DOI:** 10.1186/s13059-017-1175-5

**Published:** 2017-02-27

**Authors:** Amelia Cervera, Denisse Urbina, Marcos de la Peña

**Affiliations:** IBMCP (CSIC-UPV). C/ingeniero Fausto Elio s/n, 46022 Valencia, Spain

## Erratum

After publication of this article [1] we noticed that the funding information was incorrect. Please see the correct funding information below.

## Funding

Funding for this work was provided by the Ministerio de Economía y Competitividad of Spain and FEDER (grants BFU2011-23398 and BFU2014-56094-P). Support of the publication fee was provided by the CSIC Open Access Publication Support Initiative through its Unit of Information Resources for Research (URICI).
