# Supplementary material for: Retrozymes are a unique family of non-autonomous retrotransposons with hammerhead ribozymes that propagate in plants through circular RNAs
Source: Genome Biol. 2016 Jun 23;17:135. doi: 10.1186/s13059-016-1002-4 (PMC4918200; doi:10.1186/s13059-016-1002-4)
Supplement: Additional file 1: Table S1. — Bioinformatic compilation of putative HHRs and retrozymes found in Phytozome v10.0 and GenBank (15 Aug 2014) sequence databases of plants (see Methods section for more information). (PDF 76 kb) [file 13059_2016_1002_MOESM1_ESM.pdf]

Additional file 1.

Table 1. Bioinformatic compilation of putative HHRs and retrozymes found in Phytozome v10.0 and GenBank (Aug 15 2014) sequence databases of plants (see Materials and Methods section for more information).

Plant sp.[i]	Acc. number	# HHRs	# Retrozymes (~RNA size)
Actinidia chinensis	AONS00000000.1	2	-
Arabidopsis halleri	BASO00000000.1	13	3 (454-602 nt)
Arabidopsis lyrata subsp. lyrata	NZ_ADBK00000000.1	10	2 (567-602 nt)
Arabidopsis lyrata subsp. petraea	BASP00000000.1	10	2 (582-744 nt)
Arabidopsis thaliana[ii]	GCA_000001735	2	-
Artemisia annua[iii]	txid35608	3[vi]	1 (503 nt)
Azadirachta indica	AMWY00000000.2	108	8 (757-776 nt)
Camelina sativa	JFZQ00000000.1	6	-
Capsella rubella	ANNY00000000.1	18	8 (584-606 nt)
Chlamydomonas reindhartii	NZ_ABCN00000000.1	24[vi]	6 (~2,000 nt)
Chorispora bungeana[iii]	txid238895	4	-
Citrus clementina	AMZM00000000.1	19	13 (663-692 nt)
Citrullus lanatus	AGCB00000000.1	2	-
Citrus sinensis	AJPS00000000.1	10	11 (665-694 nt)
Citrus latifolia[iii]	txid200541	2	-
Dahlia pinnata[iii]	txid101596	5	-
Daucus carota	LNRQ00000000.1	1	-
Dianthus caryophyllus[iv]	BAUD00000000.1	1[vi], 1	Retroviroid
Eucalyptus camaldulensis	BADO00000000.1	101	26 (900-1034 nt)
Eucalyptus grandis	AUSX00000000.1	132	56 (908-1024 nt)
Fragaria ananassa	BATT00000000.1	72	8 (673-701 nt)
Helianthus argophylus[iii]	txid73275	2	1 (772 nt)
Helianthus paradoxus[iii]	txid73304	3	-
Helianthus tuberosus[iii]	txid4233	1	-
Ipomoea batatas[iii]	txid4120	1	-
Ipomoea purpurea[iii]	txid4121	5	-
Jatropha curcas	AFEW00000000.1	30	24 (693-790)
Lactuca perennis[iii]	txid43195	3	1 (432 nt)
Lactuca sativa	AFSA00000000.1	10	8 (643-652 nt)
Mangifera indica[iii]	txid29780	3	-
Manihot esculenta ssp. Flabellifolia (W14)	JPQE00000000.1	40	34 (659-739 nt)
Manihot esculenta Crantz (KU50)	JPQF00000000.1	28	9 (660-719 nt)
Erythranthe guttata	APLE00000000.1	3	-
Morus notabilis	ATGF00000000.1	2	-
Nicotiana benthamiana	CBMM000000000.1	1	-
Nicotiana tabacum	AYMY00000000.1	2[vi]	1 (641 nt)
Oryza sativa	GCA_000005425.2	2	1 (466 nt)
Pachycladon fastigiatum[iii]	txid106774	1	1 (626 nt)
Penstemon centranthifolius	JPFH00000000.1	2	1 (678 nt)
Physcomitrella patens mitDNA	NC_007945.1	2	-
Picrorhiza kurrooa[iii]	txid195120	1	-
Selaginella moellendorffii[v]	NZ_ADFJ00000000.1	111[vi]	4 (757-994 nt)
Solanum melongena	BAUE00000000.1	152	18 (664-672 nt)
Solanum torvum[iii]	txid119830	2	-
Vitis vinifera	CAAP00000000.3	7	7 (794-866 nt)

[i] Plant species not included in this table did not show the presence of HHR/retrozyme motifs at the moment of the analysis
[ii] (Przybilski et al., 2005)
[iii] Based on ESTs and Transcriptome Shotgun Assembly (TSA) sequences
[iv] (Daros and Flores, 1995)
[v] (Cervera and De la Peña, 2014)
[vi] Type I HHRs
