# Supplementary material for: Retrozymes are a unique family of non-autonomous retrotransposons with hammerhead ribozymes that propagate in plants through circular RNAs
Source: Genome Biol. 2016 Jun 23;17:135. doi: 10.1186/s13059-016-1002-4 (PMC4918200; doi:10.1186/s13059-016-1002-4)
Supplement: Additional file 2: — Schematic representation of small plant non-autonomous LTR-retrotransposons. A: TRIM (top) and SMART (bottom) retroelements. B: Truncated solo-LTR. C: Full-copy retrozyme. D: Multimeric retrozyme. LTRs are shown in blue and the approximated sizes of the different elements and regions are indicated. (PDF 86 kb) [file 13059_2016_1002_MOESM2_ESM.pdf]

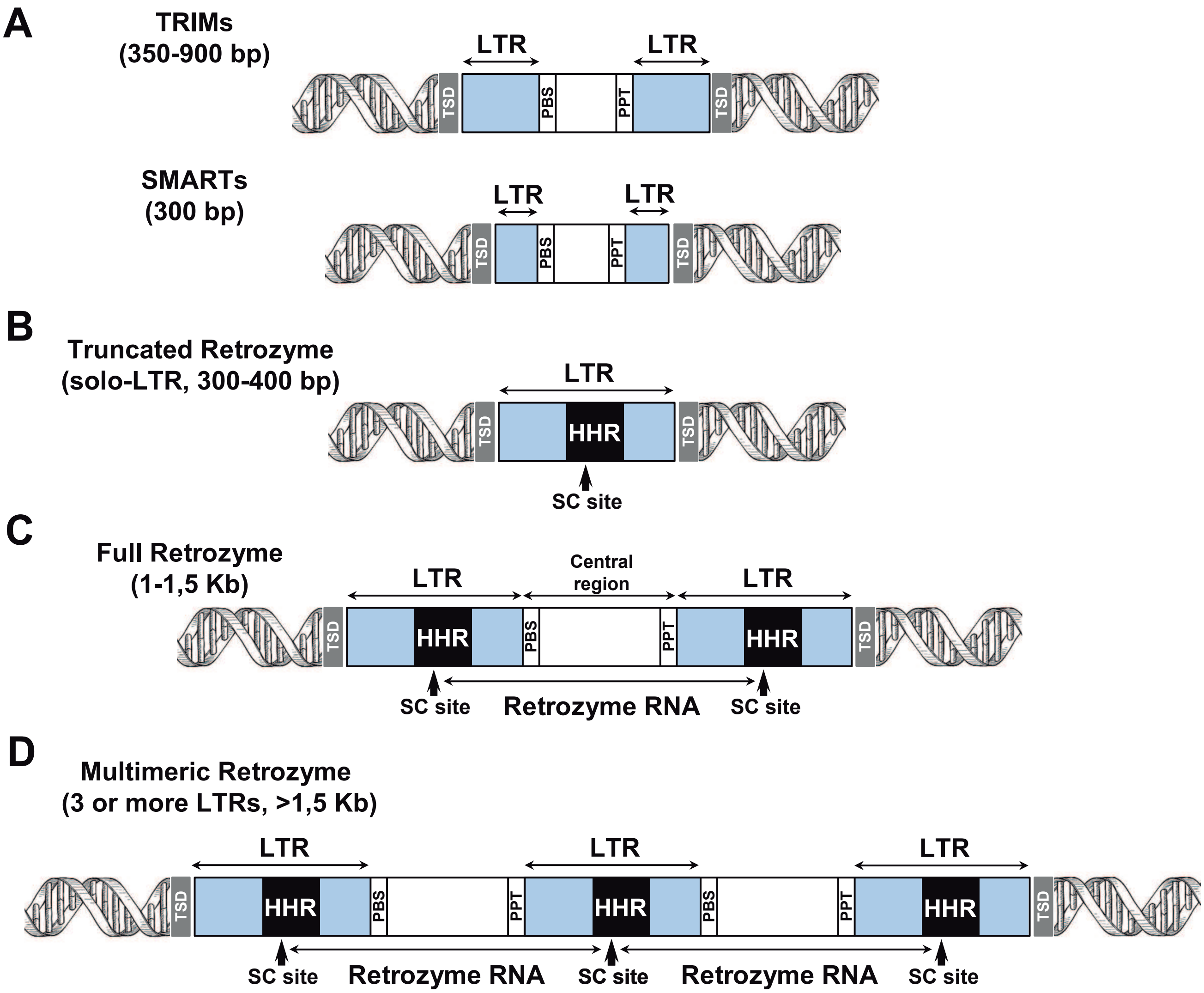

Additional file 2

Additional file 2.

Schematic representation of small plant non-autonomous LTR-retrotransposons. **a** TRIM (top) and SMART (bottom) retroelements. **b** Truncated solo-LTR. **c** Full-copy retrozyme. **d** Multimeric retrozyme. LTRs are shown in blue color and the approximated sizes of the different elements and regions are indicated.
