# Supplementary material for: Retrozymes are a unique family of non-autonomous retrotransposons with hammerhead ribozymes that propagate in plants through circular RNAs
Source: Genome Biol. 2016 Jun 23;17:135. doi: 10.1186/s13059-016-1002-4 (PMC4918200; doi:10.1186/s13059-016-1002-4)
Supplement: Additional file 3: — Hammerhead ribozymes in the genome of Jatropha curcas. A: Sequence alignment of the 30 bona fide HHRs detected in the genome of J. curcas (Bioprojects: PRJNA63485 and PRJNA279873). Only 5 out of the 30 HHRs show identical sequences, whereas the other 25 motifs showed one or more changes. B: Secondary structure of the J. curcas HHRs. Variable positions are indicated within circles (single changes) or boxes (covariations). Conserved tertiary interactions between loops 1 and 2 are shown with dotted lines. (PDF 128 kb) [file 13059_2016_1002_MOESM3_ESM.pdf]

A

B

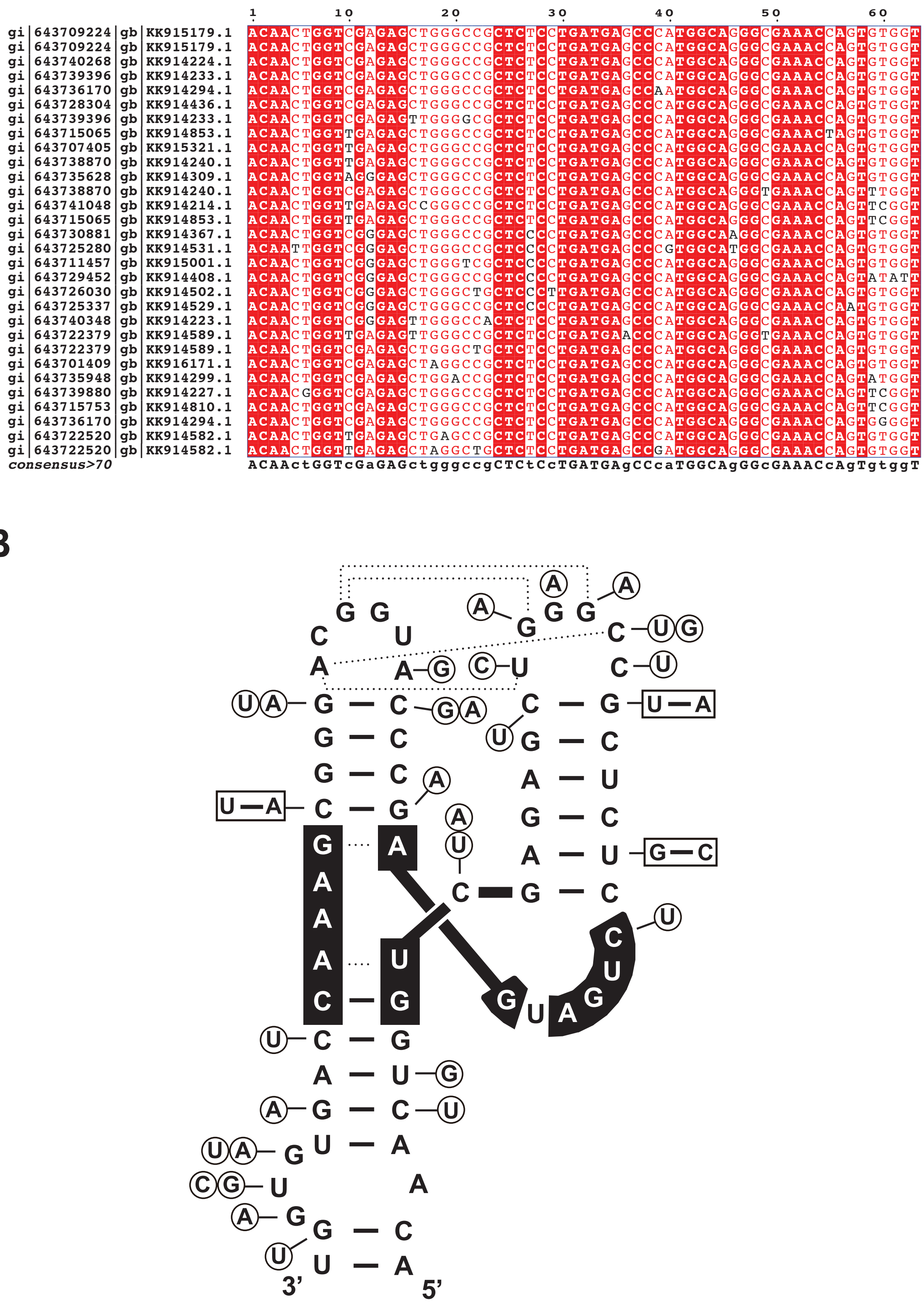

Additional file 3

Additional file 3.

Hammerhead ribozymes in the genome of *Jatropha curcas.* **a** Sequence alignment of the 30 *bona fide* HHRs detected in the genome of *J. curcas* (Bioprojects: PRJNA63485 and PRJNA279873). Only 5 out of the 30 HHRs show identical sequences, whereas the other 25 motifs showed one or more changes. **b** Secondary structure of the *J. curcas* HHRs. Variable positions are indicated within circles (single changes) or boxes (co-variations). Conserved tertiary interactions between loops 1 and 2 are shown with dotted lines.
