# Supplementary material for: Retrozymes are a unique family of non-autonomous retrotransposons with hammerhead ribozymes that propagate in plants through circular RNAs
Source: Genome Biol. 2016 Jun 23;17:135. doi: 10.1186/s13059-016-1002-4 (PMC4918200; doi:10.1186/s13059-016-1002-4)
Supplement: Additional file 4: — Catalytic functionality of J. curcas hammerhead ribozymes. Schematic representation (top) and autoradiography (bottom) of run-off transcriptions in the presence of [α-32P]-UTP of the positive and negative polarities of a genomic retrozyme fragment from Jatropha curcas. The transcribed RNAs covered approximately from position 574 to position 573 of the circular RNA depicted in Fig. 3a, and were separated by denaturing 5 % PAGE. Quantification of the bands indicated that 60 % of the transcript was processed by the ribozyme. The position of T7 and T3 RNA promoters, primer binding site (PBS), polypurine tract (PPT) and HHR self-cleavage site (arrowhead) are indicated. (PDF 108 kb) [file 13059_2016_1002_MOESM4_ESM.pdf]

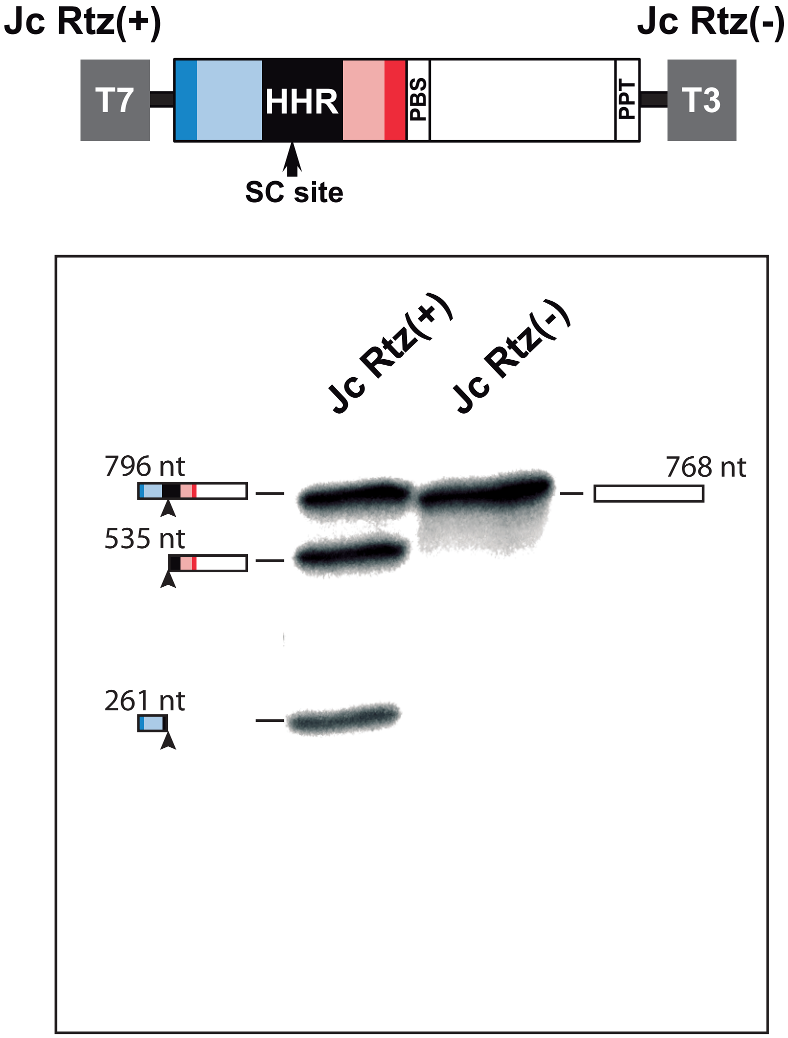

Additional file 4

Additional file 4.

Catalytic functionality of *J. curcas* hammerhead ribozymes. Schematic representation (top) and autoradiography (bottom) of run-off transcriptions in the presence of [α-32P]-UTP of the positive and negative polarities of a genomic retrozyme fragment from *Jatropha curcas*. The transcribed RNAs covered approximately, from position 574 to position 573 of the circular RNA depicted in Figure 3a, and were separated by denaturing 5% PAGE. Quantification of the bands indicated that 60% of the transcript was processed by the ribozyme. The position of T7 and T3 RNA promoters, primer binding site (PBS), polypurine tract (PPT) and HHR self-cleavage site (arrowhead) are indicated
