# Supplementary material for: Retrozymes are a unique family of non-autonomous retrotransposons with hammerhead ribozymes that propagate in plants through circular RNAs
Source: Genome Biol. 2016 Jun 23;17:135. doi: 10.1186/s13059-016-1002-4 (PMC4918200; doi:10.1186/s13059-016-1002-4)
Supplement: Additional file 5: — Circular and linear retrozyme RNAs in J. curcas and F. ananassa. A: Double PAGE analysis of an RNA extract (~20 μg) from J. curcas leaves. A gel stripe from a 5 % native polyacrylamide gel containing RNAs of 600–900 nt (left) was cut out and run on top of a second 5 % denaturing polyacrylamide gel (center). The corresponding Northern blot (right), using a digoxigenin-labelled J. curcas retrozyme fragment as a probe, revealed the presence of both circular and linear RNA forms. B: Northern blot analysis of RNA extracts (~30 μg each) from J. curcas leaves, young seedlings and seeds. Samples were run on a 5 % denaturing PAGE and were detected using the same probe as in panel A. C: An in vitro transcription of a previously cloned full genomic retrozyme of F. ananassa was run on a 5 % denaturing PAGE. The RNAs corresponding to the uncleaved full RNA (1134 nt) and double self-cleaved retrozyme RNA (679 nt) were cut out and purified (marked in red). D: Purified retrozyme RNA (679 nt) was circularized with a tRNA ligase for 2 h and run on a 5 % denaturing PAGE. The circularized RNA (upper band) was cut out and purified. E: Northern blot hybridization of a F. ananassa RNA extract (~30 μg) and previously purified RNA markers (linear, circular and uncleaved full retrozyme RNAs, ~1 ng each) run on a 5 % native PAGE. F: Northern blot hybridization of the same RNA samples as in panel E run on a 5 % denaturing PAGE. Ethidium bromide staining of the 5S rRNA is shown at the bottom as a loading control. (PDF 728 kb) [file 13059_2016_1002_MOESM5_ESM.pdf]

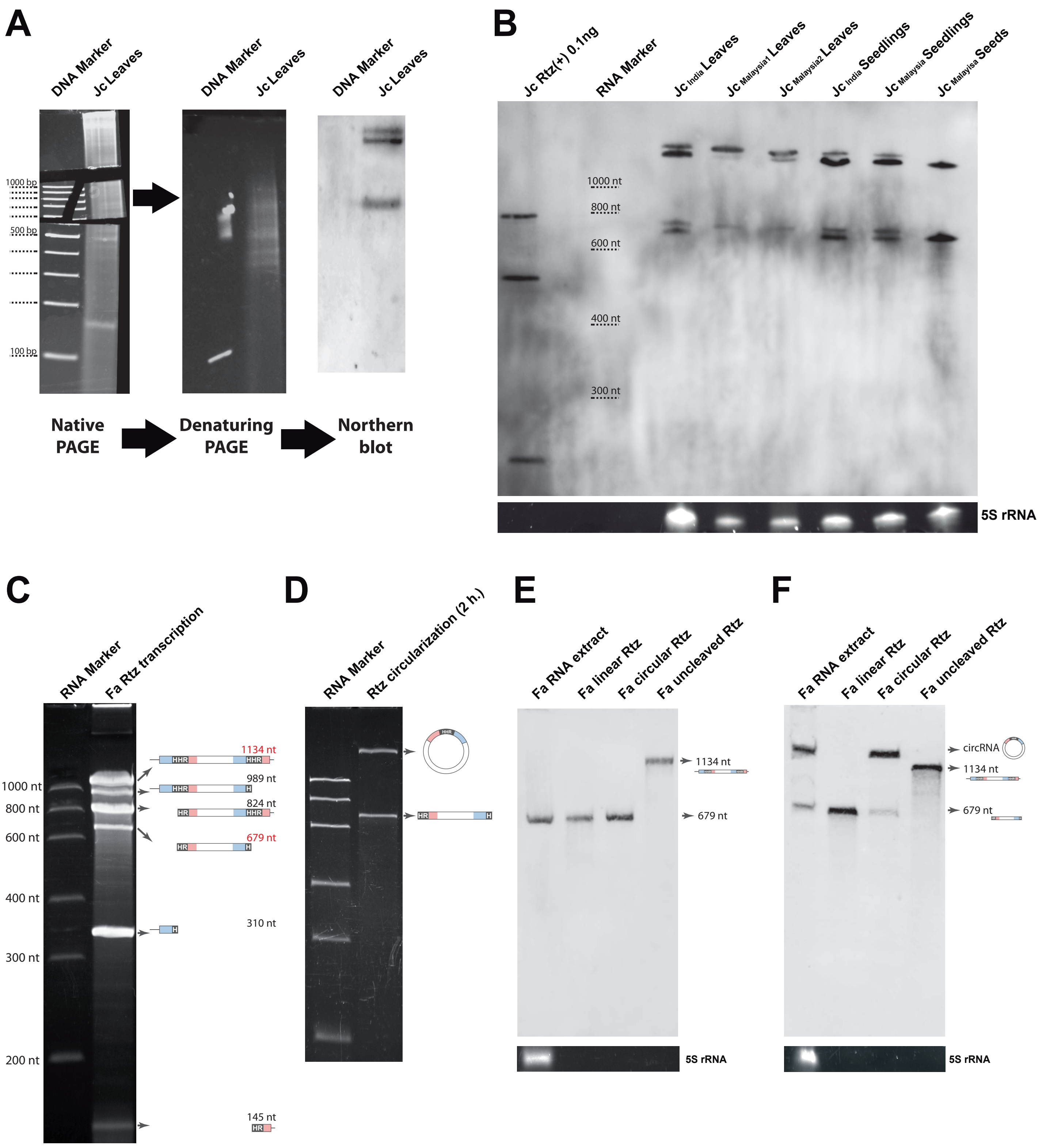

Additional file 5

Additional file 5.

Circular and linear retrozyme RNAs in *J. curcas* and *F. ananassa*. **a** Double PAGE analysis of an RNA extract (~20 µg) from *J. curcas* leaves. A gel stripe from a 5% native polyacrylamide gel containing RNAs of 600-900 nt (left) was cut out and run on top of a second 5% denaturing polyacrylamide gel (center). The corresponding Northern blot (right), using a digoxigenin-labelled *J. curcas* retrozyme fragment as a probe, revealed the presence of both circular and linear RNA forms. **b** Northern blot analysis of RNA extracts (~30 µg each) from *J. curcas* leaves, young seedlings and seeds. Samples were run on a 5% denaturing PAGE and were detected using the same probe as in panel 5a. **c** An *in vitro* transcription of a previously cloned full genomic retrozyme of *F. ananassa* was run on a 5% denaturing PAGE. The RNAs corresponding to the uncleaved full RNA (1,134 nt) and double self-cleaved retrozyme RNA (679 nt) were cut out and purified (marked in red). **d** Purified retrozyme RNA (679 nt) was circularized with a tRNA ligase for 2h and run on a 5% denaturing PAGE. The circularized RNA (upper band) was cut out and purified. **e** Northern blot hybridization of a *F. ananassa* RNA extract (~30 µg) and previously purified RNA markers (linear, circular and uncleaved full retrozyme RNAs, ~1 ng each) run on a 5% native PAGE. **f** Northern blot hybridization of the same RNA samples as in panel 5e run on a 5% denaturing PAGE. Ethidium bromide staining of the 5S rRNA is shown at the bottom as a loading control.
