# Supplementary material for: Retrozymes are a unique family of non-autonomous retrotransposons with hammerhead ribozymes that propagate in plants through circular RNAs
Source: Genome Biol. 2016 Jun 23;17:135. doi: 10.1186/s13059-016-1002-4 (PMC4918200; doi:10.1186/s13059-016-1002-4)
Supplement: Additional file 6: — Sequence variability of genomic retrozymes and retrozyme RNAs. Sequence alignment of the 24 different genomic retrozymes found in J. curcas (names in black) against 16 selected clones of circRNAs from J. curcas tissues (names in red; 8 clones for each of the two major variants detected). Only the nucleotides encompassed by the HHR self-cleavage sites (equivalent to an HHR-processed retrozyme RNA) were used in the alignment. SM and SI correspond to circRNAs from J. curcas seeds, whereas HM and HI were obtained from leaves, and PM and PI from young seedlings. Based on their highest degree of similarity with the cloned RNAs, two genomic retrozyme sequences (at the top and at the 9th position of the alignment) were chosen as the tentative parental elements. For clarity, the full sequence of the two tentative parental retrozymes is shown, whereas for all the other sequences, only the non-conserved positions are depicted. (PDF 110 kb) [file 13059_2016_1002_MOESM6_ESM.pdf]

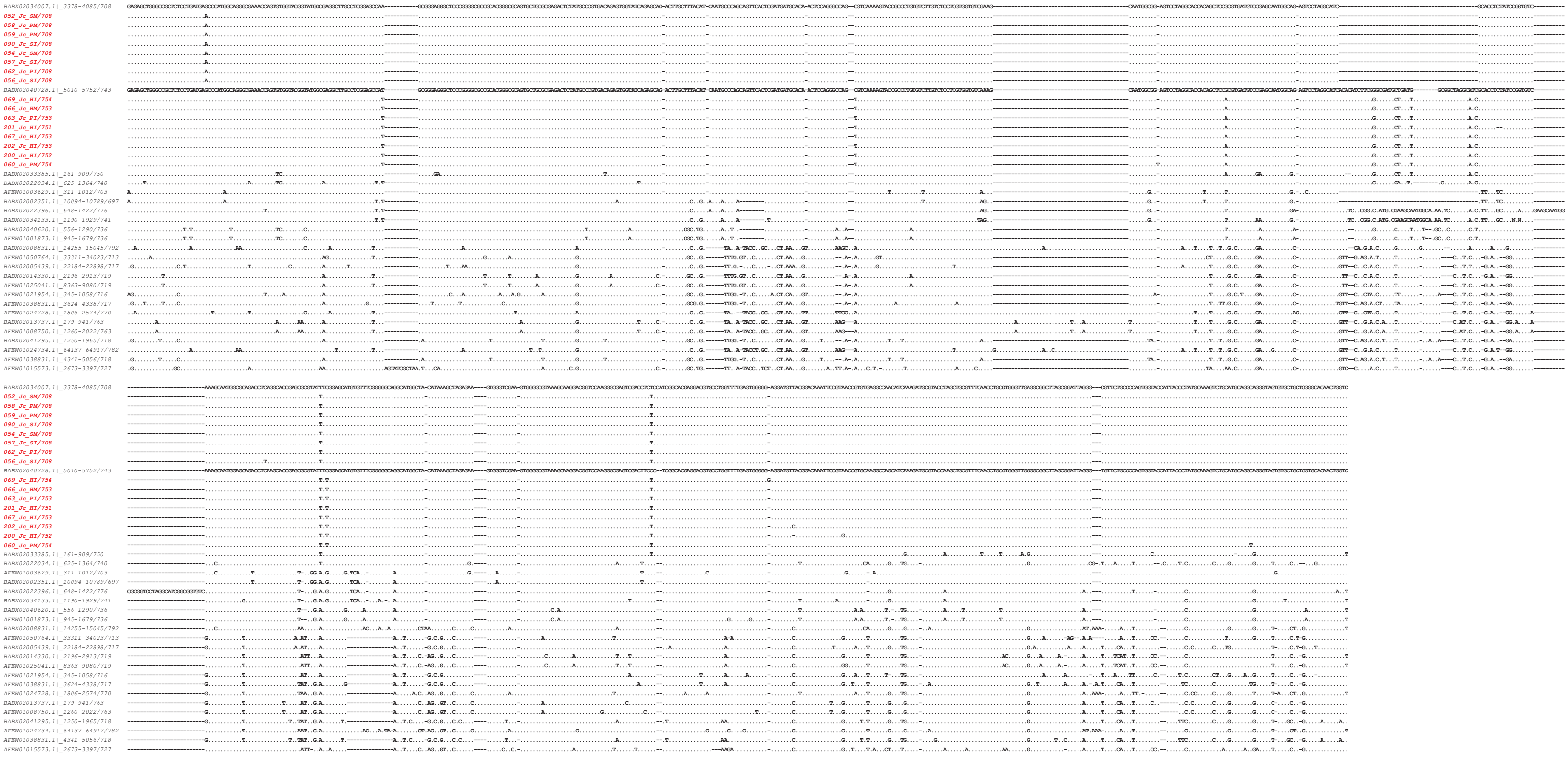

Additional file 6

Additional file 6.

Sequence variability of genomic retrozymes and retrozyme RNAs. Sequence alignment of the 24 different genomic retrozymes found in *J. curcas* (names in black) against 16 selected clones of circRNAs from *J. curcas* tissues (names in red; 8 clones for each of the two major variants detected). Only the nucleotides encompassed by the HHR self-cleavage sites (equivalent to a HHR-processed retrozyme RNA) were used in the alignment. SM and SI correspond to circRNAs from *J. curcas* seeds, whereas HM and HI were obtained from leaves, and PM and PI from young seedlings. Based on their highest degree of similarity with the cloned RNAs, two genomic retrozyme sequences (at the top and at the 9th position of the alignment) were chosen as the tentative parental elements. For clarity, the full sequence of the two tentative parental retrozymes is shown, whereas for all the other sequences, only the non-conserved positions are depicted.
