# Supplementary material for: Retrozymes are a unique family of non-autonomous retrotransposons with hammerhead ribozymes that propagate in plants through circular RNAs
Source: Genome Biol. 2016 Jun 23;17:135. doi: 10.1186/s13059-016-1002-4 (PMC4918200; doi:10.1186/s13059-016-1002-4)
Supplement: Additional file 7: — Minimum free energy secondary structure predictions for the viral satellite RNA sTRSV (top, entry M14879.1) and the viroids ELVd (middle, entry AJ536612.1) and CChMVd (bottom, entry AJ878085.1). HHR sequences are shown in purple (positive polarity) and grey (negative polarity). Self-cleavage sites are indicated with arrows. Numbering starts at the self-cleavage site of the positive polarity HHR. (PDF 86 kb) [file 13059_2016_1002_MOESM7_ESM.pdf]

sTRSV (Tobacco Ringspot Virus satellite RNA)

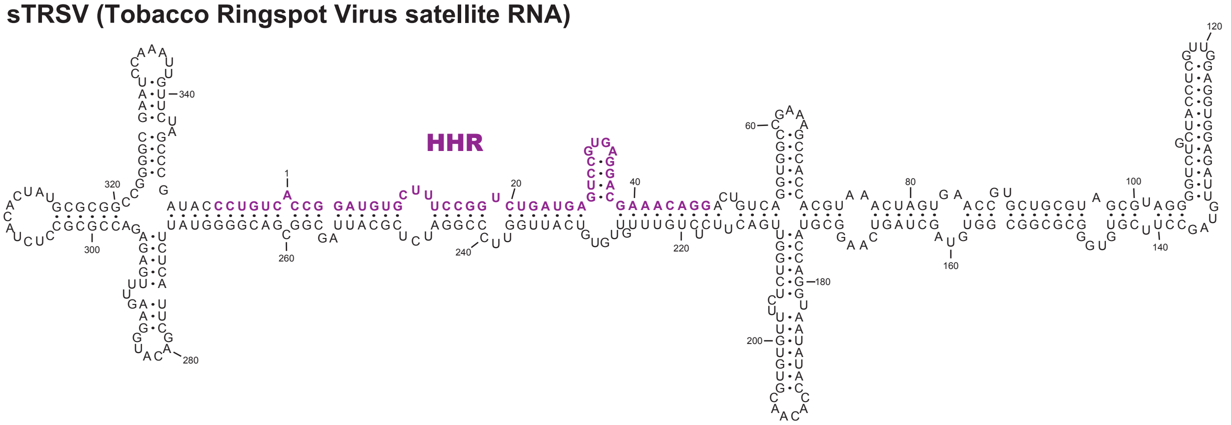

ELVd (Eggplant Latent Viroid)

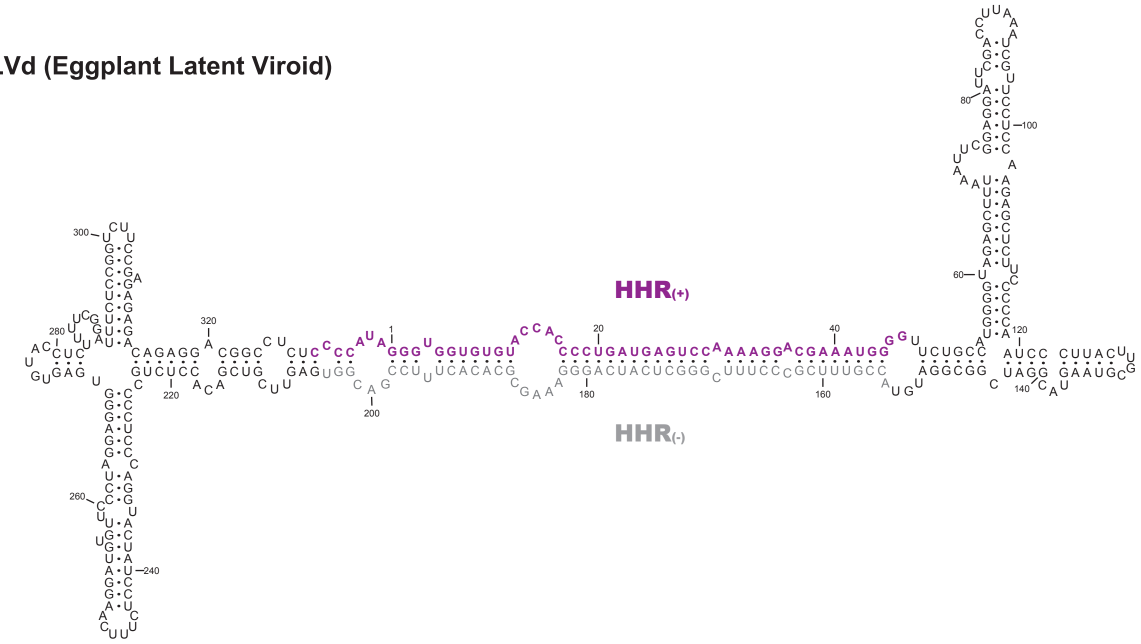

CChMVd (Chrysanthemum Chlorotic Mottle Viroid)

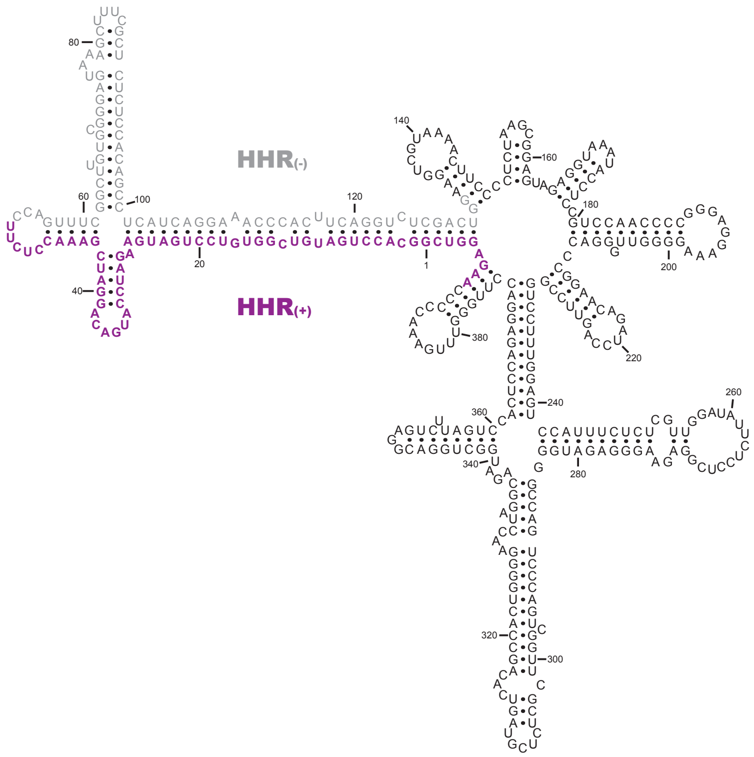

Additional file 7

Additional file 7.

Minimum free energy secondary structure predictions for the viral satellite RNA sTRSV (Top. Entry M14879.1), and the viroids ELVd (Middle. Entry AJ536612.1) and CChMVd (Bottom. Entry AJ878085.1). HHR sequences are shown in purple (positive polarity) and grey (negative polarity). Self-cleavage sites are indicated with arrows. Numbering starts at the self-cleavage site of the positive polarity HHR.
