# Supplementary material for: Retrozymes are a unique family of non-autonomous retrotransposons with hammerhead ribozymes that propagate in plants through circular RNAs
Source: Genome Biol. 2016 Jun 23;17:135. doi: 10.1186/s13059-016-1002-4 (PMC4918200; doi:10.1186/s13059-016-1002-4)
Supplement: Additional file 8: — RT-PCR amplification of negative polarity retrozyme RNAs from J. curcas and F. ananassa. A: RT-PCR experiment carried out with RNA extracts from J. curcas seeds using the direct primer Jc60D for RT to make a cDNA of the minus polarity, and the adjacent primers Jc60D and Jc60fullR (left) or Jc77D and Jc77R (right) for the PCRs (see Additional file 12). B: RT-PCR experiments performed with two different RNA extracts from F. ananassa leaves using the direct primer Fa87D for RT to make a cDNA of the minus polarity, and the adjacent primers Fa87D and Fa85R for PCR (see Additional file 12). PCR products and 100 bp DNA marker were separated by native 5 % PAGEs and stained with ethidium bromide. Controls (RT-) were the same RT-PCR experiments done without adding retrotranscriptase, whereas Controls (PCR) were PCRs done without any template. Schematic representations of a full genomic retrozyme and the positions of the oligos used for RT and PCR experiments are shown below each gel picture. (PDF 649 kb) [file 13059_2016_1002_MOESM8_ESM.pdf]

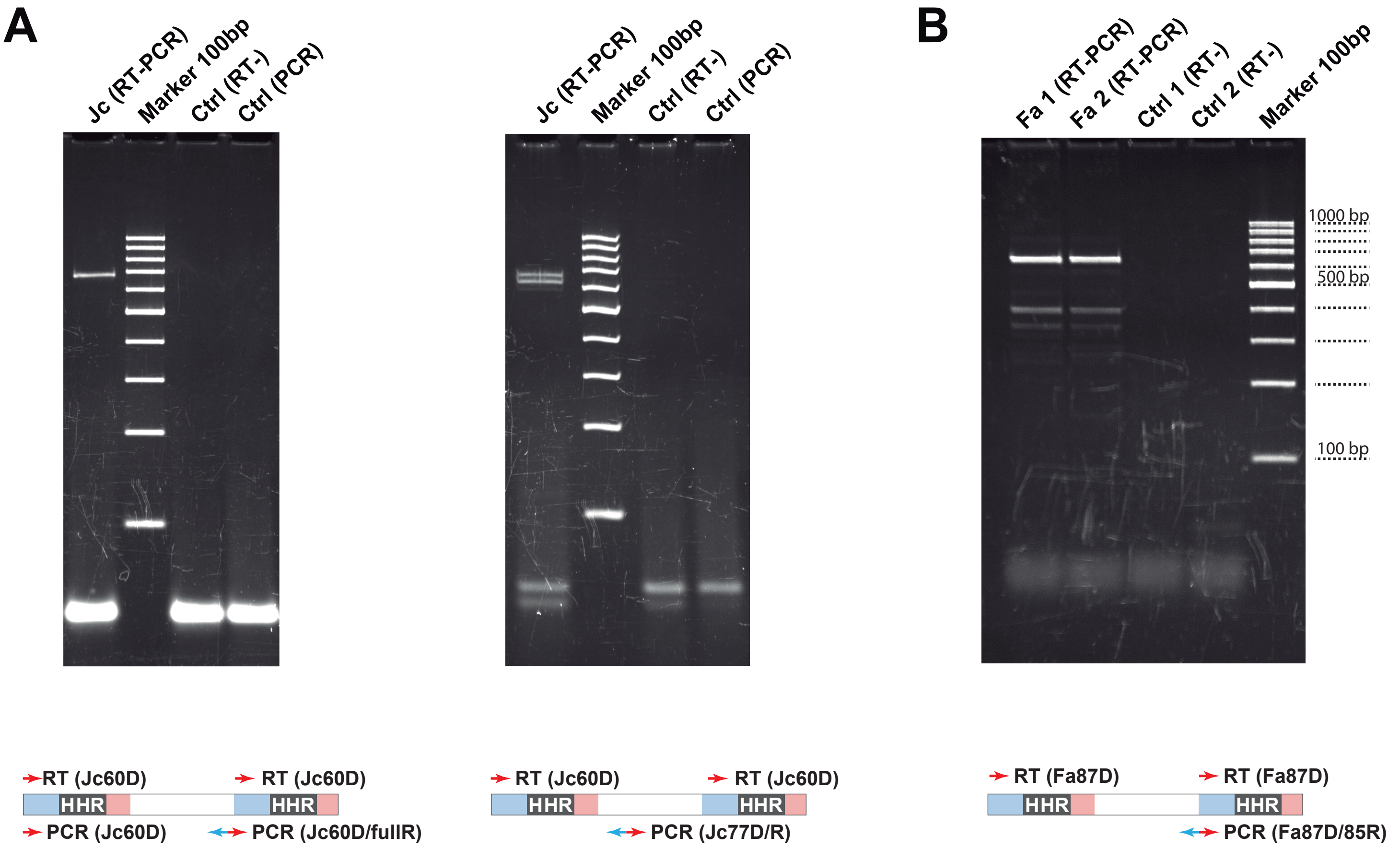

Additional file 8

Additional file 8.

RT-PCR amplification of negative polarity retrozyme RNAs from *J. curcas* and *F. ananassa*. **a** RT-PCR experiment carried out with RNA extracts from *J. curcas* seeds using the direct primer Jc60D for RT to make a cDNA of the minus polarity, and the adjacent primers Jc60D and Jc60fullR (left) or Jc77D and Jc77R (right) for the PCRs (see Additional file 12). **b** RT-PCR experiments performed with two different RNA extracts from *F. ananassa* leaves using the direct primer Fa87D for RT to make a cDNA of the minus polarity, and the adjacent primers Fa87D and Fa85R for PCR (see Additional file 12). PCR products and 100 bp DNA marker were separated by native 5% PAGEs and stained with ethidium bromide. Controls (RT-) were the same RT-PCR experiments done without adding retrotranscriptase, whereas Controls (PCR) were PCRs done without any template. Schematic representations of a full genomic retrozyme and the positions of the oligos used for RT and PCR experiments are shown below each gel picture.
