# Supplementary material for: Retrozymes are a unique family of non-autonomous retrotransposons with hammerhead ribozymes that propagate in plants through circular RNAs
Source: Genome Biol. 2016 Jun 23;17:135. doi: 10.1186/s13059-016-1002-4 (PMC4918200; doi:10.1186/s13059-016-1002-4)
Supplement: Additional file 9: — A retrozyme insertion in the genome of C. clementina is absent from the orthologous region in the genome of C. sinensis. A: Comparative analysis of two orthologous genomic regions of around 5 kb from C. clementina and C. sinensis revealed the presence in the former but not in the latter genome of a typical retrozyme element (not to scale) flanked by the target site duplications (TSDs, in grey letters). The position of hammerhead ribozymes, the primer binding site and the polypurine tract are indicated. B: Sequence alignment of the two orthologous genomic regions of C. clementina and C. sinensis. Differences between both sequences are highlighted in red. The sequence corresponding to the genomic retrozyme is underlined. (PDF 118 kb) [file 13059_2016_1002_MOESM9_ESM.pdf]

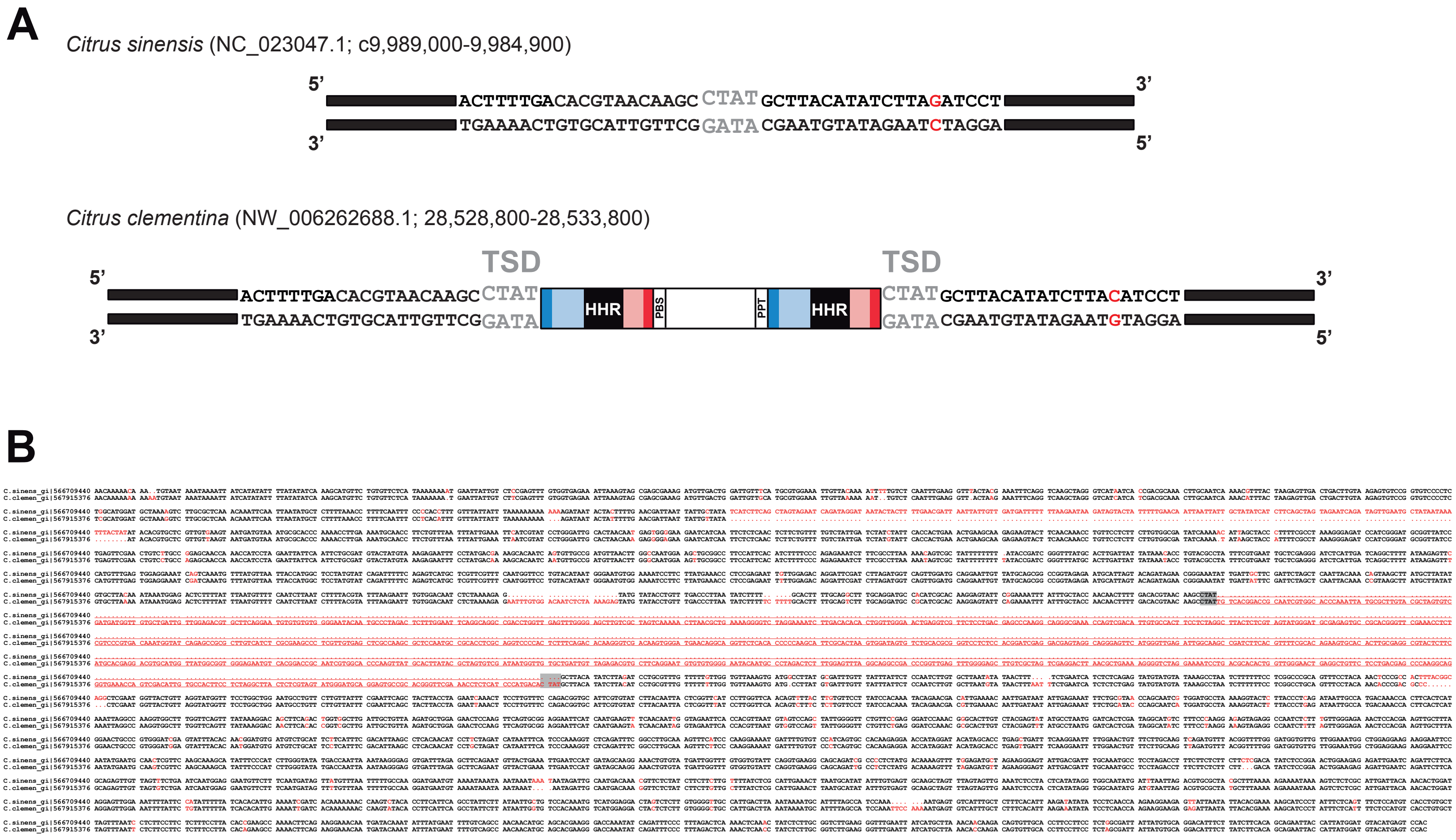

Additional file 9

Additional file 9.

A retrozyme insertion in the genome of *C. clementina* is absent from the orthologous region in the genome of *C. sinensis.* **a** Comparative analysis of two orthologous genomic regions of around 5kb from *C. clementina* and *C. sinensis* revealed the presence in the former but not in the latter genome of a typical retrozyme element (not to scale) flanked by the Target Site Duplications (TSDs, in grey letters). The position of hammerhead ribozymes, the primer binding site, and the polypurine tract are indicated. **b** Sequence alignment of the two orthologous genomic regions of *C. clementina* and *C. sinensis*. Differences between both sequences are highlighted in red color. The sequence corresponding to the genomic retrozyme is underlined.
