# Supplementary material for: Retrozymes are a unique family of non-autonomous retrotransposons with hammerhead ribozymes that propagate in plants through circular RNAs
Source: Genome Biol. 2016 Jun 23;17:135. doi: 10.1186/s13059-016-1002-4 (PMC4918200; doi:10.1186/s13059-016-1002-4)
Supplement: Additional file 10: — Minimum free energy secondary structure prediction of five plant circRNA retrozymes deduced from the corresponding genomic retrozyme sequences (GenBank entries are indicated below the species names). HHR sequences are shown in purple letters and the self-cleavage site is indicated with an arrow. Numbering starts at the self-cleavage site of the HHR. (PDF 100 kb) [file 13059_2016_1002_MOESM10_ESM.pdf]

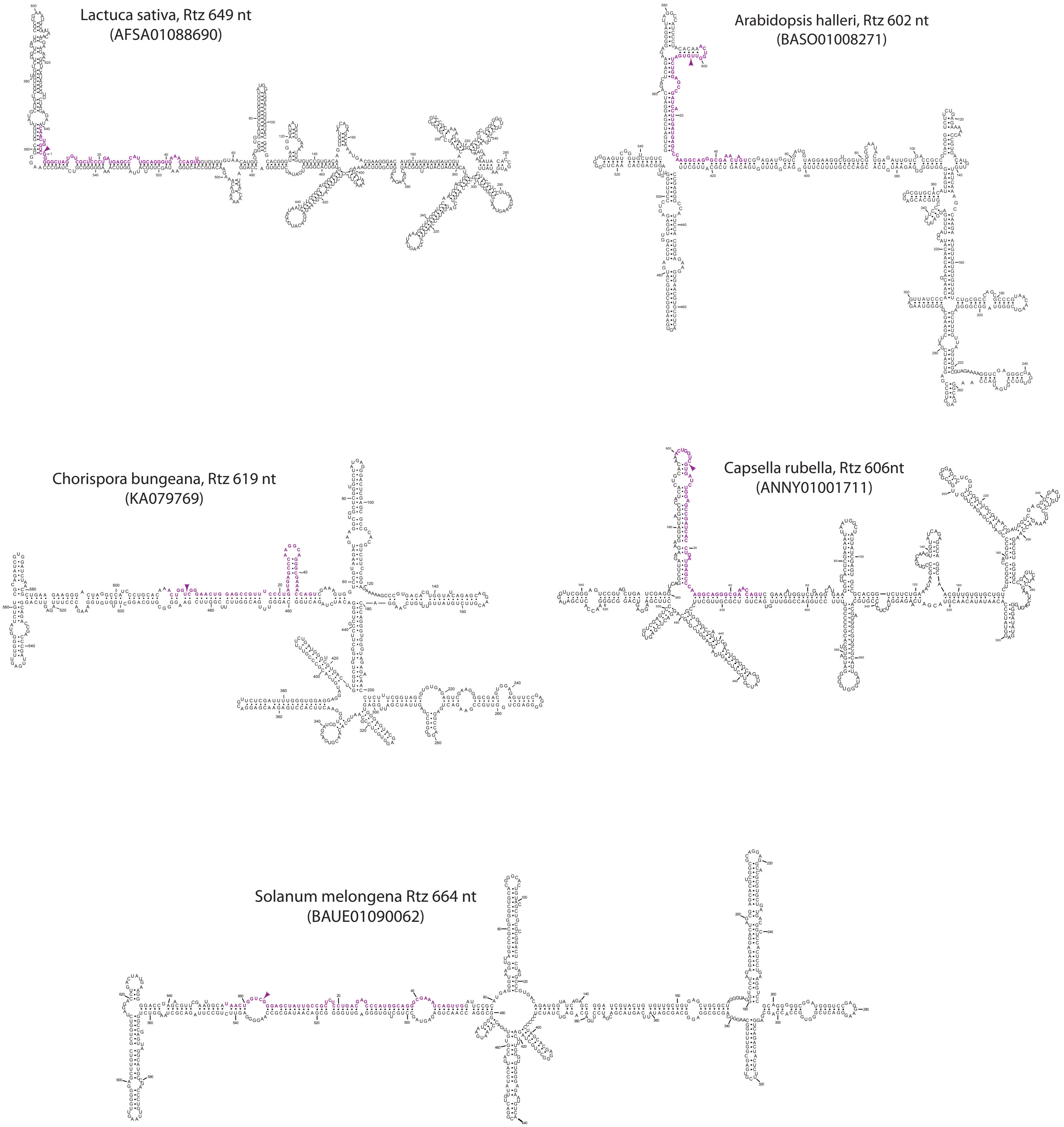

Additional file 10

Additional file 10.

Minimum free energy secondary structure prediction of five plant circRNA retrozymes deduced from the corresponding genomic retrozyme sequences (GenBank entries are indicated below the species names). HHR sequences are shown in purple letters and the self-cleavage site is indicated with an arrow. Numbering starts at the self-cleavage site of the HHR.
