# Supplementary material for: Retrozymes are a unique family of non-autonomous retrotransposons with hammerhead ribozymes that propagate in plants through circular RNAs
Source: Genome Biol. 2016 Jun 23;17:135. doi: 10.1186/s13059-016-1002-4 (PMC4918200; doi:10.1186/s13059-016-1002-4)
Supplement: Additional file 11: — Minimum free energy secondary structure prediction of putative retrozyme RNAs of metazoan genomes from different phyla, like A: rotifers, B: corals, C: arthropods, D: mollusks and E: vertebrates, deduced from the corresponding sequence repeats with HHRs. HHR sequences are shown in purple letters and the self-cleavage sites are indicated with an arrow. Numbering starts after the HHR self-cleavage site. (PDF 104 kb) [file 13059_2016_1002_MOESM11_ESM.pdf]

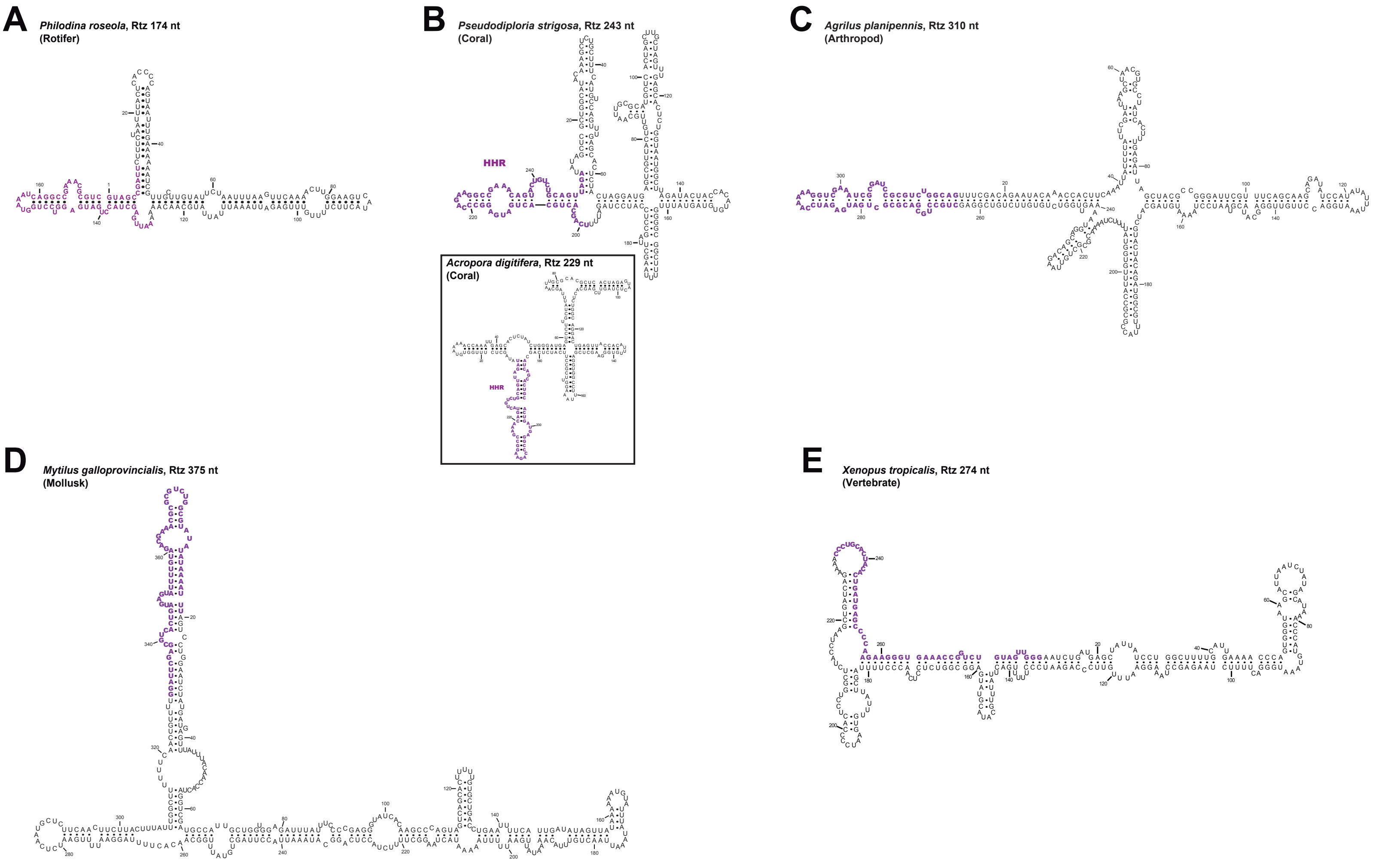

Additional file 11

Additional file 11.

Minimum free energy secondary structure prediction of putative retrozyme RNAs of metazoan genomes from different phyla, like **a** rotifers, **b** corals, **c** arthropods, **d** mollusks and **e** vertebrates, deduced from the corresponding sequence repeats with HHRs. HHR sequences are shown in purple letters and the self cleavage sites are indicated with an arrow. Numbering starts after the HHR self-cleavage site.
