# Supplementary material for: Retrozymes are a unique family of non-autonomous retrotransposons with hammerhead ribozymes that propagate in plants through circular RNAs
Source: Genome Biol. 2016 Jun 23;17:135. doi: 10.1186/s13059-016-1002-4 (PMC4918200; doi:10.1186/s13059-016-1002-4)
Supplement: Additional file 12: Table S2. — Oligonucleotide compilation. (PDF 58 kb) [file 13059_2016_1002_MOESM12_ESM.pdf]

Additional file 12.

Table 2. Oligonucleotide compilation.

Plant species	Name	Type	Primer sequence (5'-3')	Motif
Jatropha curcas	Jc60D	Forward	GGAGGATCCYACGGACAAATTCCGTRACCGTG	PPT and LTR 5' end
	Jc60FullR	Reverse	GTCTCTAGACATCCTCCCCCACTCAAAACCAG	
Jatropha curcas	Jc60AdjD	Forward	CCGTGACAGAGTGGTATCAGAGCAG	LTR 3' end and PBS
	Jc60R	Reverse	CGGGTCTAGAGTCTCGCGCAGCACTGCGCCC	
Jatropha curcas	Jc77D	Forward	TGGTCTAGAAGCAATGGCGGAGTCCTAGGCACC	Central region
	Jc77R	Reverse	CGAGGATCCAAGACACAGGGCGGTACTTTTGAC	
Fragaria ananassa	Fa87D	Forward	GAGTGTCTAGACCCGCTTTYGGGAGTGTCATYATG	PPT and LTR 5' end
	Fa85R	Reverse	CTCTCCCCCACCAAAACAAGGTGTACG	
Fragaria ananassa	Fa92D	Forward	TTGAgGATCcTTAAAAGTTGAAACTTAC	Full genomic retrozyme
	Fa92R	Reverse	CTTTTctaGAACAAAGATCATATTGTCA	(BATT01039028.1)
Eucalyptus camaldulensis	Ec72D	Forward	CACGGATCCGTGAATYCTCCCGTACYCGTGA	PPT and LTR 5' end
	Ec72R	Reverse	ACAWTCTAGACCACTCAATTCGCAACGCCCTCGTT	
Citrus sinensis	Cs70D	Forward	CTTAGGATCCTGTCACGGACYGCAATCGTGGCACCC	PPT and LTR 5' end
	Cs70R	Reverse	GTCTTCTAGATTCTCCCACCGCCATAACCATGC	
